# DRP1 mutations associated with EMPF1 encephalopathy alter mitochondrial membrane potential and metabolic programs

**DOI:** 10.1242/jcs.260370

**Published:** 2023-02-10

**Authors:** Gabriella L. Robertson, Stellan Riffle, Mira Patel, Caroline Bodnya, Andrea Marshall, Heather K. Beasley, Edgar Garza-Lopez, Jianqiang Shao, Zer Vue, Antentor Hinton, Maria S. Stoll, Sholto de Wet, Rensu P. Theart, Ram Prosad Chakrabarty, Ben Loos, Navdeep S. Chandel, Jason A. Mears, Vivian Gama

**Affiliations:** ^1^ Vanderbilt University, Cell and Developmental Biology, Nashville, TN 37232, USA; ^2^ Vanderbilt University, Molecular Physiology and Biophysics, Nashville, TN 37232, USA; ^3^ Central Microscopy Research Facility, University of Iowa, Iowa City, IA 52246, USA; ^4^ Case Western Reserve University, Department of Pharmacology and Center for Mitochondrial Diseases, Cleveland, OH 44106, USA; ^5^ Stellenbosch University, Department of Physiological Sciences, Matieland, 7602, Stellenbosch, South Africa; ^6^ Stellenbosch University, Department of Electrical and Electronic Engineering, Matieland, 7602, Stellenbosch, South Africa; ^7^ Northwestern University, Feinberg School of Medicine Department of Medicine Division of Pulmonary and Critical Care Medicine, Chicago, IL 60611, USA; ^8^ Northwestern University, Feinberg School of Medicine Department of Biochemistry and Molecular Genetics, Chicago, IL 60611, USA; ^9^ Vanderbilt University, Vanderbilt Center for Stem Cell Biology, Nashville, TN 37232, USA; ^10^ Vanderbilt University, Vanderbilt Brain Institute, Nashville, TN 37232, USA

**Keywords:** Fibroblast, DRP1, Mitochondria, Peroxisome, Cristae, Glycolysis, Oxidative phosphorylation

## Abstract

Mitochondria and peroxisomes are dynamic signaling organelles that constantly undergo fission, driven by the large GTPase dynamin-related protein 1 (DRP1; encoded by *DNM1L*). Patients with *de novo* heterozygous missense mutations in *DNM1L* present with encephalopathy due to defective mitochondrial and peroxisomal fission (EMPF1) – a devastating neurodevelopmental disease with no effective treatment. To interrogate the mechanisms by which DRP1 mutations cause cellular dysfunction, we used human-derived fibroblasts from patients who present with EMPF1. In addition to elongated mitochondrial morphology and lack of fission, patient cells display lower coupling efficiency, increased proton leak and upregulation of glycolysis. Mitochondrial hyperfusion also results in aberrant cristae structure and hyperpolarized mitochondrial membrane potential. Peroxisomes show a severely elongated morphology in patient cells, which is associated with reduced respiration when cells are reliant on fatty acid oxidation. Metabolomic analyses revealed impaired methionine cycle and synthesis of pyrimidine nucleotides. Our study provides insight into the role of mitochondrial dynamics in cristae maintenance and the metabolic capacity of the cell, as well as the disease mechanism underlying EMPF1.

## Teaser

**Highlighted Article:** Dominant-negative mutations in DRP1 identified in patients with EMPF1 result in mitochondrial and peroxisomal hyperfusion, severe alterations in mitochondrial membrane potential and metabolic reprograming.

## INTRODUCTION

Mitochondrial dynamics are controlled and executed by a vast group of proteins and other organelles. The underlying morphology of the mitochondria is an important determinant for the ability of this organelle to regulate a variety of cellular processes, such as apoptosis, cell fate, lipid biosynthesis and epigenetic modifications (Bayraktar et al., 2019; Iwata et al., 2020; Khacho et al., 2016; King et al., 2021; Prigione and Adjaye, 2011; Rasmussen et al., 2020; Vantaggiato et al., 2019). Mitochondrial fusion is primarily catalyzed by the large GTPases mitofusin 1 (MFN1) and mitofusin 2 (MFN2) at the outer mitochondrial membrane (Chen et al., 2003). The GTPase optic atrophy 1 (OPA1) is responsible for executing fusion of the inner mitochondrial membrane (Alexander et al., 2000; Delettre et al., 2000). Mitochondrial fission, which is mediated through the large GTPase dynamin-related protein 1 (DRP1; encoded by *DNM1L*), is orchestrated by a complex sequence of events (Fonseca et al., 2019; Smirnova et al., 2001). The first steps of mitochondrial fission include contact with the endoplasmic reticulum (ER) and replication of the mitochondrial genome (Friedman et al., 2011; Lewis et al., 2016). This is followed by contact and pre-constriction by actin filaments, which are associated with the ER membrane (Chakrabarti et al., 2018; Hatch et al., 2016; 2014; Ji et al., 2017, 2015; Li et al., 2015). Fission is also regulated through adaptor proteins at the outer mitochondrial membrane, including mitochondrial dynamics protein 49 (MiD49, also known as MIEF2), mitochondrial dynamics protein 51 (MiD51; also known as MIEF1), mitochondrial fission factor (MFF), and fission, mitochondrial 1 (FIS1); however, their distinct roles in fission are not completely understood (Losón et al., 2013; Osellame et al., 2016; Palmer et al., 2013). After associating with adaptor proteins, DRP1 forms a multimeric ring around the mitochondria and hydrolyzes GTP to GDP, causing a conformational change in the ring that executes fission (Chang and Blackstone, 2007; Fröhlich et al., 2013; Nagashima et al., 2020). DRP1 is essential for mitochondrial fission; without functional DRP1, the mitochondrial network becomes hyperfused. In addition to the effects on mitochondrial morphology, DRP1 also mediates the fission of peroxisomes (Koch et al., 2003; Li and Gould, 2002). Peroxisomes are highly specialized organelles vital for the β-oxidation of very-long-chain fatty acids, detoxification of reactive oxygen species (ROS), and production of cholesterol, bile acids and plasmalogens, which contribute to the phospholipid content in the brain white matter (Chandel, 2021; Ding et al., 2021; Ferreira et al., 2021; Lodhi and Semenkovich, 2014; Ravi et al., 2021; Schrader et al., 2020; Sugiura et al., 2017). The adaptor proteins MFF and FIS1 are also involved in DRP1 recruitment to sites of peroxisome fission (Gandre-Babbe and Van Der Bliek, 2008; Itoyama et al., 2013; Koch and Brocard, 2012). The contacts between mitochondria and peroxisomes are essential for linking peroxisomal β-oxidation and mitochondrial ATP generation by the electron transport chain (ETC), maintenance of redox homeostasis and lipid transfer (Chandel, 2021; Fan et al., 2016; Fransen et al., 2017). Thus, peroxisomal and mitochondrial metabolic activities are functionally interconnected. Consistent with this, peroxisomal disorders caused by mutations in genes involved in the biogenesis and function of peroxisomes, are often associated with mitochondrial dysfunction (Tanaka et al., 2019). This communication between mitochondria and peroxisomes might be particularly important during brain development and metabolic stress. However, the molecular mechanisms that facilitate this communication are still poorly understood.

**Fig. 1.**
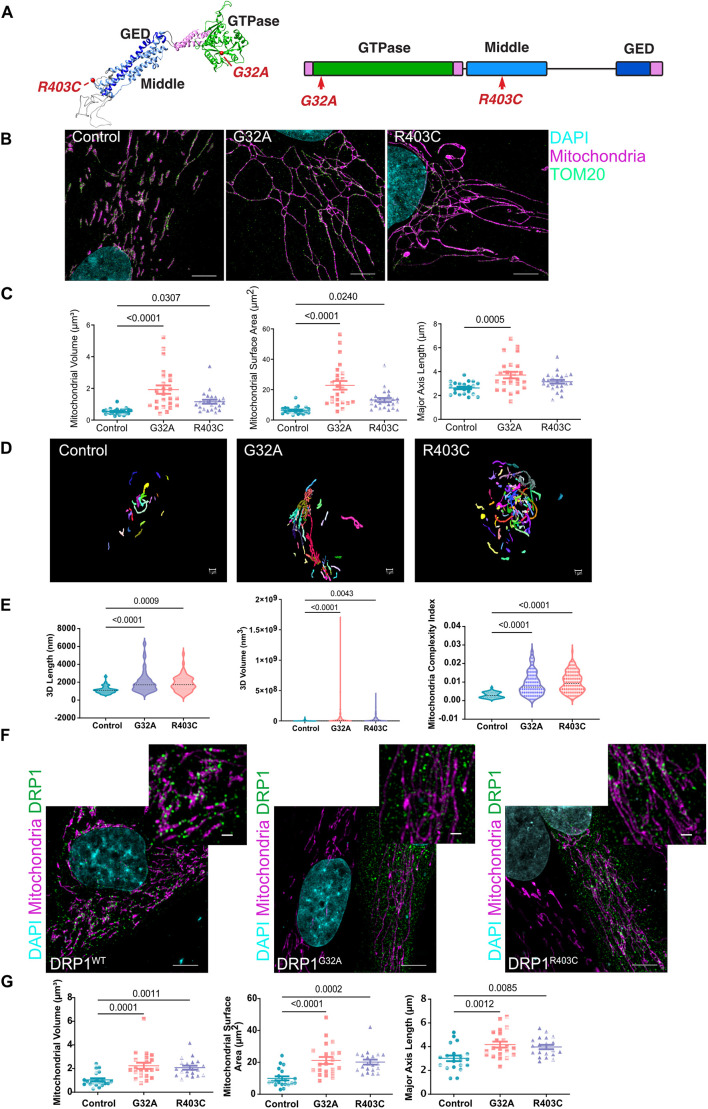
**DRP1 patient-derived fibroblasts have elongated mitochondrial morphology.** (A) Structural and schematic representation of DRP1 with mutations highlighted in red. (B) Representative maximum intensity projections of mitochondrial morphology in patient-derived fibroblasts using SIM (*n*=3 with seven cells per replicate). Scale bar: 5 μm. (C) Quantification of mitochondrial volume, surface area and major axis length. Analyzed using one-way ANOVA followed by Dunnett's multiple comparison test. (D) Representative SBF-SEM 3D reconstructed images. Each mitochondrion is colored a unique color. (E) Quantification of SBF-SEM for 3D mitochondrial length, volume and complexity index. Analyzed using one-way ANOVA followed by Dunnett's multiple comparison test and displayed in a volcano plot. (F) Representative maximum intensity projections of mitochondrial morphology in control fibroblasts transfected with either DRP1^WT^–mCherry, DRP1^G32A^–mCherry, or DRP1^R403C^–mCherry using SIM (*n*=3 with seven cells per replicate). Scale bars: 5 μm (lower) and 1 μm (upper). (G) Quantification of mitochondrial volume, surface area and major axis length. Analyzed using one-way ANOVA followed by Dunnett's multiple comparison test. Error bars shown are mean±s.e.m. and the median is indicated in the volcano plots.

In mouse models, global DRP1 knockout is embryonic lethal (Ishihara et al., 2009; Wakabayashi et al., 2009). With the advent of exome sequencing, patients with *de novo* mutations in the gene that encodes DRP1, *DNM1L*, have been identified (Chao et al., 2016; Fahrner et al., 2016; Keller et al., 2021; Liu et al., 2021; Longo et al., 2020; Nasca et al., 2016; Nolan et al., 2019; Ryan et al., 2018; Sheffer et al., 2016; Vandeleur et al., 2019; Waterham et al., 2007; Yoon et al., 2016). The disease associated with mutations in *DNM1L* is known as encephalopathy due to defective mitochondrial and peroxisomal fission-1 (EMPF1; OMIM 614388). Most reported mutations are heterozygous missense mutations. Patients present with heterogeneous symptoms, including neurodevelopmental delay, seizures, muscle abnormalities and ataxia. Patient fibroblasts show hyperfused mitochondrial networks and elevated lactate levels, suggesting an impairment in oxidative phosphorylation (Liu et al., 2021; Whitley et al., 2018). Currently, there is no effective treatment or cure for patients affected by DRP1 mutations. The life expectancy for most patients with DRP1 mutations is childhood to early adolescence (Ryan et al., 2018). Although other proteins and organelles are involved in fission, DRP1 is indispensable for mitochondrial fission (Csordás et al., 2006; Fonseca et al., 2019; Kamerkar et al., 2018; Li et al., 2015; Nagashima et al., 2020; Smirnova et al., 2001), yet the cellular consequences of DRP1 dysfunction are poorly understood. In this study, we aimed to characterize the effects of two DRP1 mutations identified in patients with EMPF1 – the G32A mutation, which lies in the catalytic GTPase domain of DRP1, and the R403C mutation, which lies in the stalk domain (Fröhlich et al., 2013) (Fig. 1A) – on mitochondrial network morphology, cristae ultrastructure and related metabolic adaptations to gain fundamental insight into the cellular adaptations to dysfunctional organelle fission and the pathophysiology of EMPF1.

Here, we resolve and quantify mitochondrial and peroxisomal morphological features associated with EMPF1-associated mutations and reveal their impact on cellular metabolism to put into perspective previously described clinical findings (Fahrner et al., 2016; Liu et al., 2021; Ryan et al., 2018). We found that the inability of DRP1 to fragment the mitochondria in patient-derived fibroblasts not only leads to the hyperfusion phenotype previously described but also to delayed cell death in response to apoptotic signals, aberrant mitochondrial cristae structure and hyperpolarized mitochondrial membrane potential. Metabolically, the changes in mitochondrial and peroxisomal morphology result in inefficiency of ETC coupling oxygen consumption with ATP production, upregulation of glycolysis and overall metabolic reprograming. Altogether, these data indicate that mitochondrial and peroxisomal fission influence the structure of the inner mitochondrial membrane, the mitochondrial membrane potential, and the metabolic state of the cell.

## RESULTS

### DRP1 variants found in patients have dominant negative effects that alter the expression of critical proteins at the mitochondria

Light microscopy studies have identified profound alterations of mitochondrial fission in cells from patients with DRP1 mutations (Ryan et al., 2018; Whitley et al., 2018). These studies highlight the clinical relevance of mitochondrial fission in neural development. We quantified the morphology of the mitochondria in two patient-derived primary fibroblast lines (one with the G32A mutation and one with the R403C mutation) at super resolution level using structured illumination microscopy (SIM). Both patient fibroblast lines showed increased mitochondrial volume, surface area and major axis length, consistent with a hyperfused mitochondrial morphology, as reported for DRP1-null cells (Fonseca et al., 2019; Kamerkar et al., 2018; Smirnova et al., 2001) (Fig. 1B,C). Live-cell imaging of mitochondria stained with MitoTracker confirmed that mitochondrial fission events were rare in patient cells compared to in control fibroblasts (Fig. S1A). High-resolution three-dimensional (3D) images of control and patient fibroblasts were obtained using serial block facing-scanning electron microscopy (SBF-SEM) (Garza-Lopez et al., 2021). Manual segmentation and stack reconstruction of the mitochondrial networks validated the increased length and volume of the mitochondrial networks, as well as increased mitochondrial complexity index (MCI) in patient fibroblasts (Fig. 1D,E). The MCI is a quantitative measurement of network complexity, which considers both volume (V) and surface area (SA) of the mitochondria (MCI=SA^3^/16π^2^V^2^) (Faitg et al., 2021; Vincent et al., 2019). Both DRP1 mutations resulted in significant alterations of the mitochondrial network with G32A fibroblasts showing slightly more severe elongation than R403C fibroblasts (Fig. 1E; Fig. S1B).

In patients, both DRP1 mutations are heterozygous (Fahrner et al., 2016; Ryan et al., 2018; Whitley et al., 2018), suggesting a dominant effect that can interfere with the ability of wild-type DRP1 to fragment the mitochondria. To test this, we generated mCherry-tagged constructs containing the patient-derived mutations and expressed them in human fibroblasts homozygous for endogenous wild-type DRP1. Overexpression of mCherry–DRP1-G32A and mCherry–DRP1-R403C by transient transfection in wild-type fibroblasts demonstrated that, as previously described (Montecinos-Franjola et al., 2020, Fahrner et al., 2016; Ryan et al., 2018; Whitley et al., 2018), the mitochondrial hyperfusion seen in patient cells is not due to the genetic background and that these mutations act in a dominant negative manner (Fig. 1F,G). Both DRP1 mutations significantly impaired DRP1 function in human fibroblasts.

**Fig. 2.**
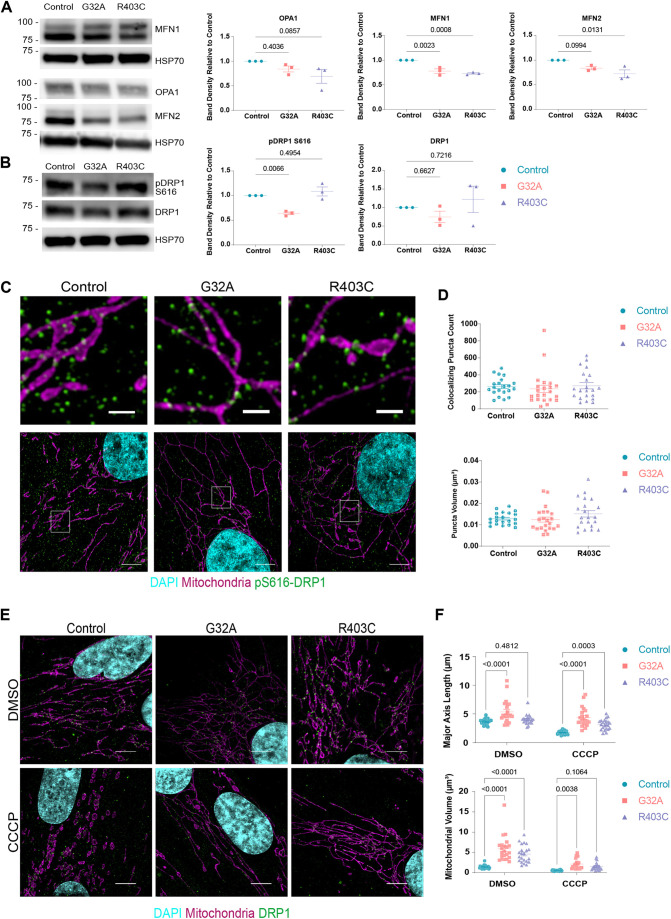
**Mutant G32A and R403C DRP1 are recruited to mitochondria.** (A) Western blot of total protein lysate isolated from patient fibroblasts and probed for mitochondrial fusion proteins. Representative image of three independent biological replicates. Band density is normalized to loading control (HSP70) and relative to control cells. (B) Western blot of total protein lysate isolated from patient fibroblasts and probed for mitochondrial fission proteins. Band density is normalized to loading control (HSP70) and relative to control cells. Representative image of three independent biological replicates. (C) Representative maximum intensity projections of mitochondrial and pDRP1 (Ser616) in patient-derived fibroblasts using SIM (*n*=3 with seven cells per replicate). Top image is a zoom of white box from bottom image. Scale bars: 5 μm (lower) and 1 μm (upper). (D) Quantification of pDRP1 (Ser616) puncta that overlap with mitochondria and volume of pDRP1 puncta (Ser616). Analyzed using one-way ANOVA followed by Dunnett's multiple comparison test. (E) Representative maximum intensity projections of mitochondrial morphology in patient-derived fibroblasts treated with either DMSO or CCCP for 2 h using SIM (*n*=3 with seven cells per replicate). Scale bar: 5 μm. (F) Quantification of mitochondrial volume, surface area and major axis length. Analyzed using one-way ANOVA followed by Dunnett's multiple comparison test. Error bars shown are mean±s.e.m.

Hyperfusion of the mitochondrial membrane due to sequestration and inactivation of DRP1 is thought to lead to unopposed fusion events that increase the severity of mitochondrial elongation (Palmer et al., 2013). We examined the effects of DRP1 mutations on the protein expression levels of other mitochondrial dynamics proteins. MFN1 was expressed at significantly lower levels in both patient cells than in control cells (Fig. 2A). However, this difference was not significant at the mRNA level (Fig. S1C). MFN2 expression both at the protein and mRNA level was significantly lower in R403 but not in G32A fibroblasts (Fig. 2A; Fig. S1C). There was no significant difference in the expression of OPA1 (Fig. 2A). Therefore, the lack of active fission appears to have an effect on the overall expression of mitofusins at the outer mitochondrial membrane.

Phosphorylation of DRP1 at serine 616 has been proposed to be a critical determinant of DRP1 recruitment and activity (Koch and Brocard, 2012; Taguchi et al., 2007; Valera-Alberni et al., 2021). Whether mutations in DRP1 found in patients with EMPF1 affect the levels of phosphorylation at serine 616 has not been explored. We examined DRP1 levels in patient-derived fibroblasts. Although there was no significant difference in total DRP1 expression, G32A patient cells expressed significantly less pS616-DRP1 than control cells (Fig. 2B). Given that G32A mutation impairs DRP1 phosphorylation to a greater extent than the R403C mutation, the GTPase domain of DRP1 might be structurally or functionally involved in DRP1 phosphorylation. We wondered whether the expression of one copy of mutant DRP1 in patient fibroblasts affected the recruitment of endogenous pS616-DRP1 to the mitochondria. To test this, we quantified the recruitment of phosphorylated DRP1 to the outer mitochondrial membrane using super-resolution microscopy. On average, there was no significant difference in the recruitment of pS616-DRP1 to the mitochondria in patient cells and control cells (Fig. 2C,D). In all cell lines, pS616-DRP1 was found in close contact to the mitochondrial membrane. Because recruitment of phosphorylated DRP1 does not distinguish mutant DRP1 from wild-type (WT) DRP1, we performed live-cell imaging of overexpressed mCherry-tagged DRP1 WT, G32A or R403C to examine the dynamics of the mutant forms of DRP1 as they are recruited to the outer mitochondrial membrane. Although all forms of DRP1 were recruited to the mitochondria, the live-cell imaging experiments revealed an overall decrease in the time that the mutant forms of DRP1 spend at the mitochondrial membrane (Fig. S2). As this defect of the mutant proteins was only detected in the live-cell imaging experiments, the possibility that this phenotype might only become evident under conditions of stress (phototoxicity during live cell imaging in this case), cannot be excluded. In the future, it would be informative to determine whether the mutant dimers are recruited to the mitochondria and sequester the WT forms of the protein, explaining the dominant-negative effects. Establishing the kinetics of association and dissociation of the mutant and WT forms of DRP1 in both homeostatic and stressed conditions could provide additional insight into the mechanism behind these phenotypes. These results point to generalized defects in the kinetics of mutant DRP1 that result in stalling of the fission events in patient fibroblasts.

The onset of apoptosis is accompanied by mitochondrial remodeling, stable association of DRP1 to the mitochondrial membrane and increased fragmentation of the mitochondrial membrane (Martinou and Youle, 2006; Wasiak et al., 2007). In DRP1-deficient cells, this remodeling of mitochondria is significantly reduced, and the onset of cell death is delayed (Frank et al., 2001; Lee et al., 2004). To determine the effects of mitochondrial stress on mitochondrial fragmentation in patient fibroblasts, we quantified changes in mitochondrial fission in response to carbonyl cyanide m-chlorophenyl hydrazone (CCCP) treatment. As previously shown using RNAi experiments in HeLa cells, patient fibroblasts showed reduced mitochondrial fragmentation induced by CCCP, compared with control cells (Fig. 2E,F). Although some mitochondrial fragmentation might occur, mitochondrial volume and major axis length were significantly larger in CCCP-treated patient cells than in control cells. Upon apoptosis, BAX translocates to the mitochondria and cytochrome *c* is released to the cytosol (Smaili et al., 2001); however, in DRP1-depleted cells BAX is recruited to the mitochondria and cytochrome *c* release is delayed. To examine apoptosis in these patient cells, we treated the cells with apoptosis-inducing drugs with different mechanisms of action, including DNA damage (etoposide), mitochondrial uncoupling (CCCP), and pan-kinase inhibition (staurosporine). Patient fibroblasts show higher cell survival when treated with CCCP and etoposide; as well as lower caspase-3 activity than control fibroblasts when treated with staurosporine (STS) (Fig. S3A,B). Interestingly, basal levels of both BAX and BAK (also known as BAK1) are significantly reduced in patient fibroblasts with DRP1 mutations (Fig. S3C). Levels of the anti-apoptotic protein MCL-1 were significantly reduced in G32A fibroblasts but not in R403C fibroblasts, while BCL-XL (encoded by *BCL2L1*) expression levels remained relatively constant in all cell lines. Thus, patient fibroblasts might display a compensatory adaptation to reduce the basal levels of BAX and/or BAK activation. Whether this reduction in the ability to undergo apoptosis in response to mitochondrial stress is a widespread phenomenon in all tissues of EMPF1 patients and/or whether apoptosis inhibition results in deleterious accumulation of mitochondrial and nuclear DNA-encoded mutations remains unexplored. No significant alteration in the protein expression of DRP1 adaptors was detected (Fig. S3D).

### Mitochondrial hyperfusion in DRP1 mutant fibroblasts leads to higher glycolytic rate and increased mitochondrial membrane potential

**Fig. 3.**
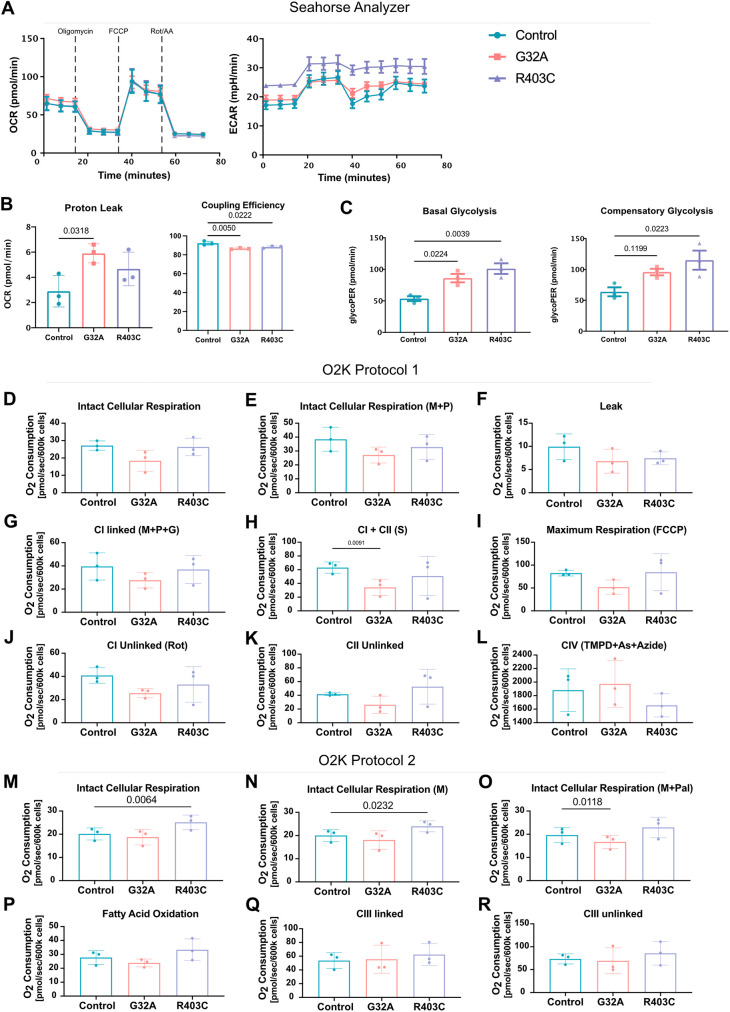
**Mitochondrial defects lead to inefficient coupling of the electron transport chain and higher glycolytic rate.** (A) Oxygen consumption rate (OCR) and extracellular acidification rate in patient fibroblasts as measured using the Seahorse mitochondrial stress test (*n*=3 with 20 wells per replicate). Oligomycin applied at 20 min, FCCP applied at 40 min, and rotenone and antimycin A (Rot/AA) applied at 60 min (indicated on graph). Analyzed using two-way ANOVA followed by Šídák's multiple comparisons test. (B) Proton leak and coupling efficiency calculated using OCR. (C) Basal glycolysis of patient fibroblasts measure using the Seahorse glycolytic rate assay. Compensatory glycolysis measured following application of rotenone and antimycin A. (D–L) O_2_ consumption rate of intact cells followed by isolated complex O_2_ consumption using protocol 1. Analyzed using one-way ANOVA followed by Dunnett's multiple comparison test. Malate (M), pyruvate (P), glutamate (G), succinate (S), trifluoromethoxy carbonylcyanide phenylhydrazone (FCCP), rotenone (Rot), tetramethyl-p-phenylenediamine (TMPD) and ascorbate (As). (M–R) O_2_ consumption rate of intact cells followed by isolated complex O_2_ consumption using protocol 2. Analyzed using one-way ANOVA followed by Dunnett's multiple comparison test. Malate (M), palmitoylcarnitine (Pal). Error bars shown are mean±s.e.m.

Mitochondrial morphology is tightly linked to metabolic function (Dorn, 2015; Wai and Langer, 2016); thus, we assessed the effects of DRP1 patient mutations on cellular metabolism by measuring rates of glycolysis and oxidative phosphorylation (OxPhos) in cells using the sensitive high-throughput Seahorse XF Analyzer. The Seahorse analyzer measures extracellular oxygen consumption as a readout of OxPhos, and extracellular acidification as a readout of glycolysis. Basal and maximal oxygen consumption was not significantly different in mutant cells compared to control, suggesting no major defects in ATP production (Fig. 3A). However, proton leak was significantly higher in the G32A patient cells, and coupling efficiency was significantly lower in both patient lines (Fig. 3B). Therefore, although basal ATP production is not perturbed in the mutant cells, the ETC is not effectively coupling substrate oxidation and ATP synthesis. The extracellular acidification rate was significantly increased in both patient lines, consistent with an upregulation of glycolysis (Fig. 3A) and that hyperlactacidemia is observed in more than 50% of patients with DRP1 mutations (Liu et al., 2021). A glycolytic rate assay confirmed that both basal and compensatory glycolysis were higher in both patient lines (Fig. 3C). Thus, although the cells can produce ATP, DRP1 mutations cause a defect in OxPhos efficiency and a slight metabolic dependency on glycolysis (Fig. 3C; Fig. S4A).

Given the deficiency in OxPhos efficiency, we sought to dissect functional differences in individual ETC complexes in mitochondria when comparing control and mutant fibroblasts. Therefore, as a complement to our Seahorse analysis, we performed high-resolution respirometry using the Oxygraph-2k (O2k, Oroboros Instruments, Austria) to measure OxPhos within intact permeabilized cells (Ye and Hoppel, 2013). Oxygen consumption rates (OCRs) were measured in the presence of several selective substrates and inhibitors to measure the contributions of distinct ETC complexes. In protocol 1, the activity of complexes I, II and IV (CI, CII and CIV) was sequentially assessed and measured. First, intact cellular respiration (ICR) was determined to obtain a baseline rate prior to the addition of any substrates. Consistent with the Seahorse analyzer results, the DRP1-mutant fibroblasts showed equivalent baseline OxPhos to that in control fibroblasts (Fig. 3D). Then, substrates for CI, malate and pyruvate were added (Fig. 3E), and digitonin was added to permeabilize the cell membrane and allow malate and pyruvate to enter the cell for oxidation, which reveals the leak rate in the absence of ADP. In this assay, there was not a significant increase in proton leak between control and mutant fibroblasts when substrates were not limited (Fig. 3F).

Based on our results from the Seahorse assay, where the major difference was decreased coupling efficiency in mutant fibroblasts compared with control fibroblasts, we next examined the functionality of individual ETC complexes. In protocol 1, ADP was first added to stimulate respiration of coupled mitochondria (also known as state 3; Hsiao and Hoppel, 2018; Ye and Hoppel, 2013). This was followed by addition of glutamate, another CI substrate, to assess CI OxPhos. Succinate, a CII substrate, was then added to measure the coupled CI plus CII OxPhos. Although we found no significant differences in the CI capacity (Fig. 3G), the DRP1 G32A fibroblasts showed a significant decrease in the coupled CI plus CII OxPhos (Fig. 3H). The uncoupler, trifluoromethoxy carbonylcyanide phenylhydrazone (FCCP), was then added to assess the maximal oxidative capacity, and we observed a slight decrease, albeit not significant, in the DRP1 G32A fibroblasts (Fig. 3I). Next, the addition of rotenone, a CI inhibitor, allowed us to determine the uncoupled CI rate, and the remaining activity could be attributed to CII. No significant differences were detected for CI and CII when mitochondria were uncoupled, so the complexes did not exhibit a defect (Fig. 3J,K). To assess complex IV OxPhos, we added tetramethyl-p-phenylenediamine and ascorbate, which donates electrons to cytochrome *c*, followed by azide, a CIV inhibitor; no differences in CIV activity were detected (Fig. 3L). Thus, our assessment of complexes I, II and IV in the mutant G32A fibroblasts revealed a potential deficiency in the linked CI plus CII mitochondrial OxPhos. This decrease in linked CI plus CII activity is likely due to the additive effect of combined rates given that both CI and CII trended to lower activity on their own. This decrease in CI and CII activity was not seen in the R403C fibroblasts.

A second method, protocol 2, was performed in parallel for each cell line to measure the OxPhos for fatty acid oxidation (FAO) and complex III (CIII). The ICR rate was first assessed (Fig. 3M) followed by the addition of malate and palmitoylcarnitine, a long-chain fatty acid derivative (Fig. 3N,O). Under these conditions, we found some differences in the ICR of DRP1 R403C mutant fibroblasts (Fig. 3M,N) that were not detected by protocol 1 or Seahorse analysis. We also detected a decrease in the ICR of DRP1 G32A fibroblasts upon addition of malate and palmitoylcarnitine (Fig. 3O). Upon permeabilization with digitonin, ADP was then added to assess the state 3 rate of palmitoylcarnitine oxidation; no significant overall differences in the rate of FAO were detected (Fig. 3P). Next, we added rotenone to assess any non-mitochondrial oxidation, and duroquinol (DHQ), a CIII substrate, was introduced to measure the coupled CIII rate (Fig. 3Q). Finally, FCCP was again added to determine the uncoupled CIII rate. There were no differences in the coupled CIII activity or the uncoupled CIII oxidation. Collectively, our results showed that there is impaired coupled CI plus CII activity in G32A but not in R403C fibroblasts (Fig. 3D–Q; Fig. S4B,C). Still, these differences are relatively mild, suggesting that these cells have adapted to overcome metabolic limitations associated with impaired mitochondrial fission.

With the observed upregulation of glycolysis with no accompanied increase in OxPhos, we sought to interrogate a potential impairment in pyruvate transport. Two proteins are essential for mitochondrial pyruvate transport in humans, mitochondrial pyruvate carrier 1 and 2 (MPC1 and MPC2) (Bricker et al., 2012; Herzig et al., 2012). MPC1 and MPC2 associate to form an ∼150-kDa complex in the inner mitochondrial membrane. Real-time quantitative PCR (RT-qPCR) gene expression analysis showed a significant downregulation of *MPC2* expression in R403C cells and *MPC1* in both patient lines (Fig. S5A). These data suggest that the upregulation in glycolysis seen in DRP1 mutant patient fibroblasts is accompanied by impaired pyruvate import into the mitochondria. We also examined the expression of MPC1 and MPC2 using western blotting, which revealed a significant decrease in MPC2 expression in the R403C cells (Fig. S5B).

**Fig. 4.**
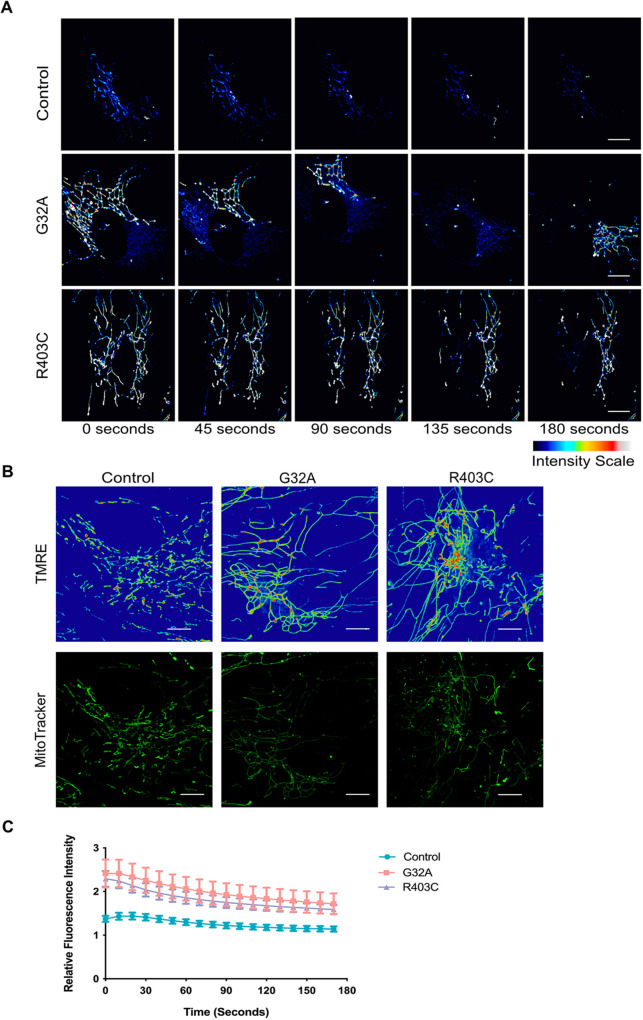
**DRP1 patient fibroblasts have more hyperpolarized mitochondrial membrane potential.** (A) Representative maximum intensity projections of live-cell imaging of patient-derived fibroblasts treated with 500 nM TMRE for 10 min. Cells were imaged every 15 s for 15 min using spinning disk confocal (*n*=3 with seven cells per replicate). (B) Representative maximum intensity projections of live-cell imaging of patient-derived fibroblasts treated with 500 nM TMRE for 10 min and 100 nM MitoTracker Green for 20 min. Cells were imaged every 10 s for 3 min using spinning disk confocal (*n*=3 with 7–10 cells per replicate). (C) Quantification of total fluorescent intensity of TMRE relative to MitoTracker Green over time. Error bars shown are mean±s.e.m. Scale bars: 5 µm.

The major contributor of the proton motive force of the ETC is the mitochondrial membrane potential, which drives OxPhos. Thus, changes in coupling efficiency can point to perturbation of the mitochondrial membrane potential and the maintenance of key ion gradients. We investigated this possibility using the fluorescent sensor tetramethylrhodamine ethyl ester (TMRE). TMRE is a cell-permeant, cationic, red-orange and fluorescent dye that is sequestered by polarized mitochondria. We found that mitochondrial membrane potential, as reported by TMRE intensity relative to MitoTracker, was significantly increased in patient fibroblasts (Fig. 4A–C), indicative of hyperpolarization of the mitochondria. In addition to the overall increase in fluorescent intensity, the hyperpolarization in mutant cells was very dynamic and moved throughout the cell over time (Fig. 4A). These data indicate that mitochondrial membrane potential is impacted by fission dynamics and altered in DRP1 mutant cells.

### DRP1 mutations alter mitochondrial cristae morphology

**Fig. 5.**
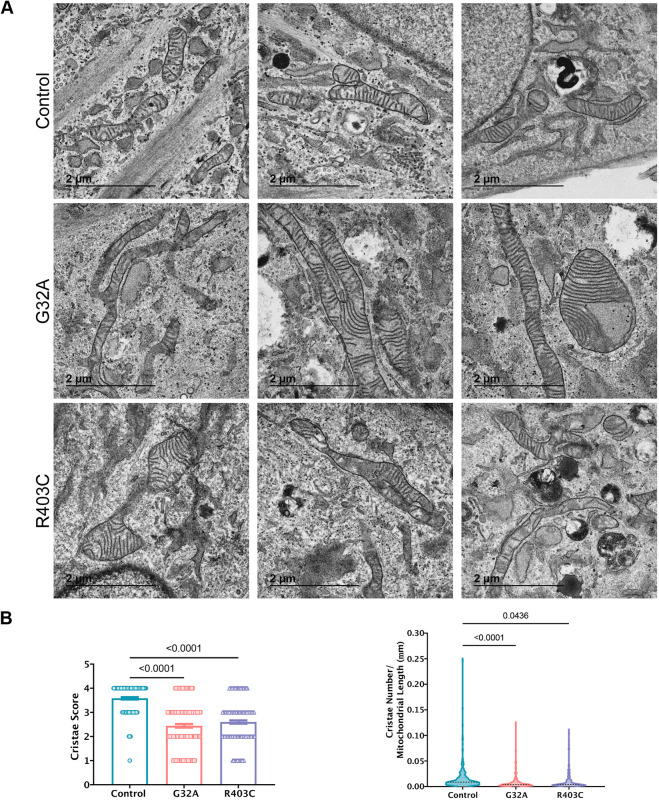
**Cristae morphology is altered in patient cells.** (A) TEM of patient fibroblasts. Three representative images per line are shown. (B) Quantification of cristae from TEM images for cristae score and cristae number relative to mitochondrial length. Analyzed using one-way ANOVA followed by Dunnett's multiple comparison test. Error bars shown are mean±s.e.m. (left) and the median is indicated in the volcano plot (right).

Cristae topology readjustments participate in energy balance control (Eramo et al., 2020; Kondadi et al., 2020). The hyperpolarization of the mitochondrial membrane detected in patient fibroblasts led us to assess the effects of DRP1 mutations on cristae morphology. We used a previously optimized method to quantify and analyze cristae morphology changes from transmission electron microscopy (TEM) (Eisner et al., 2017; Lam et al., 2021). In this method, cristae abundance is calculated by manually quantifying the number of cristae per mitochondrion, and a score between 0 (worst) and 4 (best) is assigned based on the morphology [0, no well-defined cristae; 1, no cristae in more than ∼75% of the mitochondrial area; 2, no cristae in ∼25% of mitochondrial area; 3, many cristae (over 75% of area) but irregular; 4, many regular cristae] (Fig. S5C). TEM of patient fibroblasts confirmed the mitochondrial elongation observed in SIM (Fig. S5D–G) and revealed differences in the cristae structure (Fig. 5A). The quantification of cristae morphology demonstrated a reduced cristae score and decreased cristae number relative to mitochondrial length in DRP1-deficient cells compared with what was seen in the control cells (Fig. 5B). These data link aberrant mitochondrial membrane fission caused by DRP1 mutations to generalized cristae structural changes, as previously reported with the loss of DRP1 expression (Ban-Ishihara et al., 2013). The structure and respiratory function of mitochondrial cristae are maintained by the mitochondrial contact sites and cristae organizing system (MICOS), a large complex of scaffolding and membrane-shaping proteins localized at the inner mitochondrial membrane. Given the reduced cristae score in DRP1-deficient cells, we examined expression of some key components of the MICOS complex. *MIC19*, *MIC25* and *MIC60* (also known as *CHCHD3*, *CHCHD6* and *IMMT*, respectively) are all reduced in either one or both patient lines at the mRNA level; however, protein expression is not significantly changed (Fig. S5H). Thus, the structural abnormalities seen in patient fibroblasts might not be a result of changes in the expression of MICOS proteins. Additional biochemical assays are needed to assess the functionality of MICOS proteins in the context of EMPF1.

### Patient fibroblasts display hyperfused peroxisomes

**Fig. 6.**
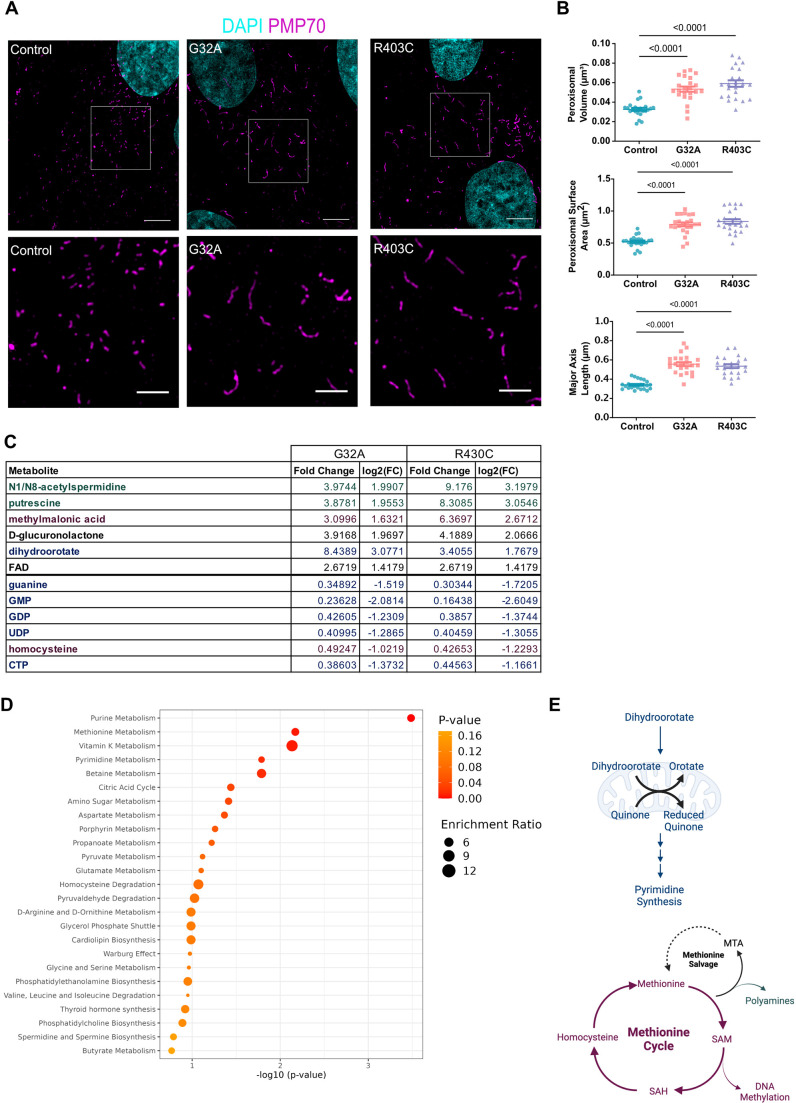
**Patient fibroblasts have elongated peroxisomes and altered metabolic profiles.** (A) Representative maximum intensity projections of peroxisomal morphology in patient-derived fibroblasts using SIM (*n*=3 with 7 cells per replicate). Lower image is a zoom of the boxed area indicated in the top image. Scale bars: 5 μm (upper) and 1 μm (lower). (B) Quantification of peroxisomal volume, surface area and major axis length. Analyzed using one-way ANOVA followed by Dunnett's multiple comparison test. Error bars shown are mean±s.e.m. (C) Table displays fold change of metabolites that were significantly different from control in both G32A and R403C. (D) Pathway enrichment analysis was performed based on all metabolites that were significantly different than control. (E) Schematic of pathways significantly altered in both G32A and R403C. Color coded to illustrate the pathways represented by metabolites (in C).

In addition to catalyzing mitochondrial fission, DRP1 also participates in peroxisomal fission (Kamerkar et al., 2018). Although fission of peroxisomes has been demonstrated to be involved in peroxisomal growth and division, the functional consequences of peroxisomal fission are not completely understood. Previous reports using EMPF1 patient fibroblasts have not reported elongation of peroxisomes, but DRP1-null cells have been reported to have hyperfused peroxisomes (Kamerkar et al., 2018). To interrogate the effects of DRP1 mutations on peroxisome morphology, we performed super-resolution microscopy. SIM revealed that both mutations in DRP1 result in significant elongation of peroxisomes (Fig. 6A). Quantification of the peroxisome structure revealed an increase in volume, surface area and length of peroxisomes (Fig. 6B), demonstrating that DRP1 mutations found in patients with EMPF1 result in impaired peroxisome fission. Then, we assessed the recruitment of DRP1 to peroxisomes by colocalization. Surprisingly, recruitment of DRP1 to peroxisomes appeared severely impaired in the R403C patient fibroblasts, compared with control and G32A cells (Fig. S6A,B). In contrast to DRP1 recruitment to the mitochondria, mutation in the stalk domain of DRP1 appears to cause a defect in peroxisomal recruitment.

Given that peroxisomes and mitochondria are hubs for the regulation of fatty acid metabolism, we first tested whether the dramatic changes in morphology would alter the expression of key genes involved in fatty acid oxidation in patient fibroblasts. The expression of these increased in a DRP1 phosphomimetic mouse model (Valera-Alberni et al., 2021). RT-qPCR analysis showed that the expression of carnitine palmitoyltransferase I (*CPT1*) and acyl-coA dehydrogenase medium chain (*ACADM*) are significantly lower in patient cells, compared with the control (Fig. S6C). CPT1 is associated with the outer mitochondrial membrane and is responsible for catalyzing the transfer of the acyl group of acyl-CoA to L-carnitine. Fatty acids are then able to move from the cytosol into the inner membrane space of the mitochondria and the matrix, where fatty acid oxidation occurs. *ACADM* encodes for the medium-chain-acyl-CoA dehydrogenase (MCAD) protein, which catalyzes the first step of mitochondrial fatty acid beta-oxidation. Thus, the reduction in *CPT1* and *ACADM* expression might hinder the ability of the mitochondria to carryout fatty acid oxidation.

To examine the ability of patient fibroblasts to perform fatty acid oxidation, we used the Seahorse analyzer to quantify OCRs of patient fibroblasts treated with either vehicle or etomoxir, a CPT1 inhibitor. The cells were cultured with minimal glucose and glutamine and high L-carnitine and palmitate to promote fatty acid oxidation over glycolysis or glutaminolysis. Control fibroblasts treated with etomoxir show a reduction in maximal respiration following FCCP injection. Both G32A and R403C patient-derived cells grown under minimal glucose/ glutamine and high L-carnitine and palmitate showed a significant decrease in OCR (Fig. S6D,E). However, treatment with etomoxir, compared to the vehicle, showed no further decrease in maximal respiration following FCCP injection (Fig. S6D,E). These data indicate that in contrast to the reliance of control cells on long-chain fatty acid oxidation under conditions of maximal respiration, both patient lines have adapted to the inhibition of long-chain fatty acid import into mitochondria under these conditions. All together these data suggest that cells with DRP1 mutations have impaired spare respiratory capacity when reliant solely on fatty acid oxidation (Fig. S6D,E). Furthermore, DRP1 mutant fibroblasts appear to have compensatory adaptations to alterations in long-chain fatty acid oxidation. The extent of this adaptation appears to be dependent on which domain of DRP1 the mutation is located and could be mechanistically correlated to the expression of key mitochondrial enzymes involved in fatty acid oxidation and/or peroxisomal function.

### Metabolic dysregulation in DRP1 mutant fibroblasts

Our observations that DRP1 mutations affect fatty acid oxidation are not surprising considering the critical role that mitochondrial and peroxisomal morphology have in this metabolic pathway (Houten and Wanders, 2010; Longo et al., 2016). Nonetheless, whether DRP1 mutations could have a more general effect on the metabolic profile of these cells has not been investigated. To explore this, we performed metabolomic profiling of patient fibroblasts using ultra-high-performance liquid chromatography and high-resolution mass spectrometry and tandem mass spectrometry (UHPLC-MS/MS). Metabolomic analysis showed 12 different metabolites that were significantly dysregulated in both DRP1 mutant fibroblasts, compared with the control (Fig. 6C). Pathway analysis of the dysregulated metabolites show pyrimidine metabolism as a top hit (Fig. 6D). Specifically, dihydroorotate is higher whereas the levels of many downstream metabolites in the pyrimidine synthesis pathway are decreased. These data suggest a block in pyrimidine synthesis, which might be upstream of quinone reduction. Pyrimidine nucleotides are essential precursors for nucleic acid synthesis and are involved in polysaccharide and phospholipid biosynthesis, detoxification processes, and protein and lipid glycosylation (Fig. 6E) (Fumagalli et al., 2017). In addition, mutant fibroblasts appear to have an aberrant methionine cycle, a series of metabolic reactions in which the essential sulfur-containing amino acid methionine is catabolized and recycled (Fig. 6E) (Lauinger and Kaiser, 2021). Both methylmalonic acid and homocysteine are dysregulated in patient fibroblasts (Fig. 6C). The methionine cycle is tightly linked to polyamine synthesis, which is also altered in patient cells as shown by the changes in N1/N8-acetylspermidine and putrescine. An elevated activity of polyamine metabolism, which directly branches from the methionine cycle, has long been associated with rapid cell proliferation, but the mechanisms underlying their modes of action have not been defined.

### Mitochondrial hyperfusion is rescued by MFN2 silencing

**Fig. 7.**
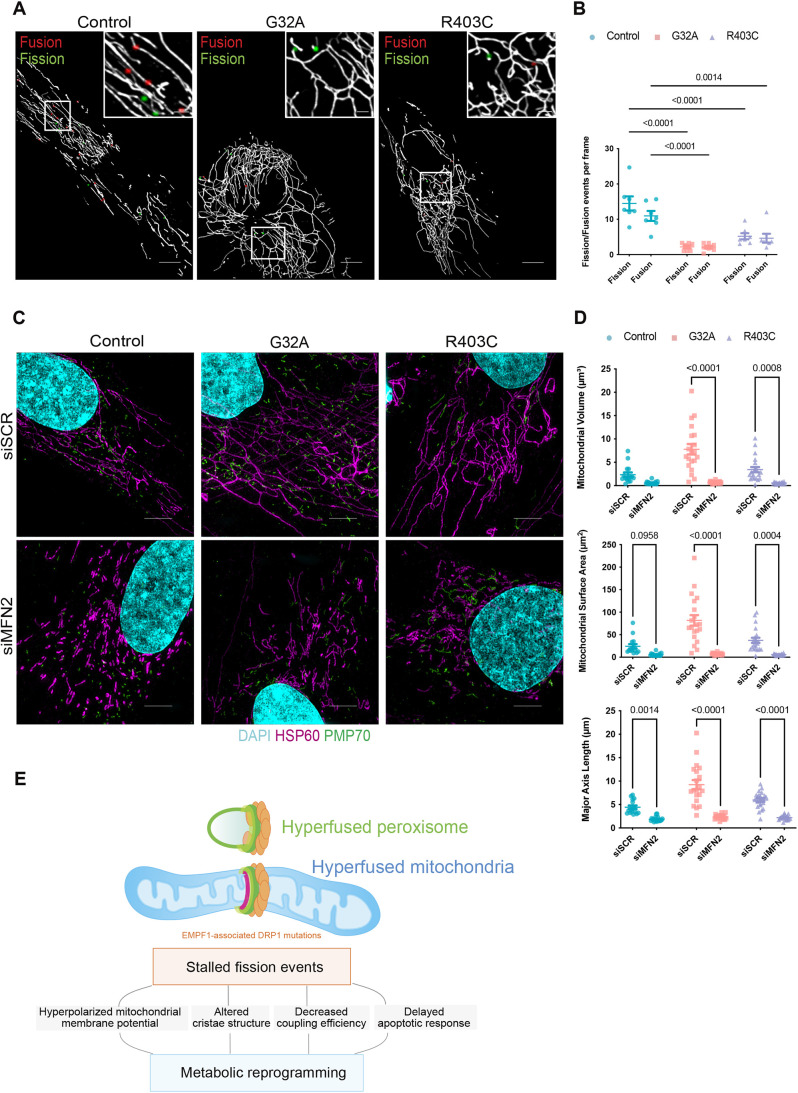
**Current model of the cellular responses to EMPF1-associated DRP1 mutations.** (A) Representative images of live-cell timelapse imaging of mitochondrial dynamics in patient-derived fibroblasts treated with Mitotracker Green with fission events marked in green and fusion events marked in red. Cells were imaged every 10 s for 3 min using spinning disk confocal (*n*=3 with 7 cells per replicate) Scale bar: 5 μm (main images); 1 μm (inset). (B) Quantification of mitochondrial dynamics using MEL. Analyzed using one-way ANOVA followed by Dunnett's multiple comparison test. (C) Representative maximum intensity projections of mitochondrial and peroxisomal morphology in patient-derived fibroblasts treated with either siRNA against MFN2 (siMFN2) or scrambled siRNA (siSCR) using SIM (*n*=3 with 7 cells per replicate). Scale bars: 5 μm. (D) Quantification of mitochondrial volume, surface area, and major axis length. Analyzed using one-way ANOVA followed by Dunnett's multiple comparison test. Error bars shown are mean±s.e.m. (E) Summary schematic. EMPF1-associated mutations do not appear to alter the capacity of DRP1 to be recruited to points of fission, but rather cause stalling of the fission events. Pausing peroxisomal and mitochondrial fission results in alterations of cristae structure, changes in mitochondrial membrane potential, decreased mitochondrial coupling efficiency, delayed cell death response, and overall metabolic adaptations.

The exact mechanisms by which the mitochondrial fusion machinery is positioned to sites of fusion are not completely understood. However, fission and fusion events occur in rapid succession (Liu et al., 2009), and fission and fusion machineries localize in close proximity at the mitochondria–ER membrane contact sites (Abrisch et al., 2020). Given the intricate relationship between these opposing dynamics, we examined the rates of fission and fusion in live cells to determine whether defects in fission could impact fusion rates. Our analysis in live cells showed that, in control cells, the number of fission and fusion events per frame are very similar, supporting the concept that these dynamic events are in equilibrium and constantly occurring in tandem to one another under physiological conditions. Consistent with this phenomenon, in patient cells, the reduced number of fission events was accompanied by a concomitant decrease in the number of fusion events (Fig. 7A,B). Thus, defective fission leads to fewer fusion events contributing to a less dynamic mitochondrial network phenotype in patient cells.

The deleterious effects of fission deficiency can be restored by deletion of the mitochondrial fusion machinery (Chen et al., 2015). To further determine the contribution of fusion to the mitochondrial network phenotype, we tested whether downregulation of MFN2 would rescue the mitochondrial hyperfusion phenotype in patient fibroblasts. Indeed, MFN2 downregulation restored all the parameters of mitochondrial morphology altered by DRP1 mutations (Fig. 7C,D; Fig. S6A), whereas the peroxisomal elongation was not restored (Fig. S6B). Collectively, these results show that the mitochondrial hyperfusion phenotype can be rescued by modulating the levels of mitochondrial fusion.

## DISCUSSION

DRP1 is an essential gene for embryonic development (Ishihara et al., 2009; Wakabayashi et al., 2009). RNAi-based approaches in human cell culture models have been used to explore the impact of DRP1 loss on mitochondrial fission and downstream processes, such as apoptosis and mitophagy (Kleele et al., 2021; Otera et al., 2016; Smirnova et al., 2001). Although loss of DRP1 results in mitochondrial hyperfusion, it is not fully understood how DRP1 mutations can impact cellular metabolic function, cristae structure and/or peroxisomal function. The analysis of organelle morphology in fibroblast lines derived from patients with EMPF1 that harbor one WT copy of DRP1 and one missense copy of DRP1, demonstrate that mutant DRP1 can have a dominant-negative effect that results in stalling of the fission machinery, which has a wide impact on cellular function (Fig. 7E).

Mitochondrial fission is essential for cellular processes, such as apoptosis, mitophagy and mitochondrial biogenesis. However, how mitochondrial fission helps maintain mitochondrial ultrastructure and/or cellular metabolism have not been thoroughly explored. Our studies revealed that although DRP1 is recruited to the mitochondria in EMPF1 patient cell lines, the fission event is incomplete, and thus, mitochondrial fission is paused. This stalling of mitochondrial fission results in changes to cristae morphology – notably a decrease in cristae score and number – indicative of immature and poorly formed cristae. Changes in redox signaling can lead to cysteine oxidation of MIC60 (Li et al., 2021; Wasilewski and Chacinska, 2021), an inner mitochondrial membrane protein responsible for cristae structure integrity. Thus, the lack of proper mitochondrial fission in patient cells might disrupt redox signaling, which might be responsible for the aberrant cristae morphology detected in patient cells. Detailed metabolic analysis of patient fibroblasts showed that these cells have decreased coupling efficiency and upregulation of glycolysis, suggesting that the functional impact of stalled fission is ETC inefficiency. This inefficiency may be due to the changes in cristae structure, since cristae structure is essential for F_0_F_1_-ATPase to function effectively (Afzal et al., 2021; Cogliati et al., 2013; Davies et al., 2011; Wilkens et al., 2013). Furthermore, the maximal OCR of the mutant cells when relying mainly on fatty acid oxidation is diminished. These data might be attributed to changes in CPT1 expression and/or mitochondrial or peroxisomal morphology. Very little is known about how peroxisomal morphology impacts β-oxidation of fatty acids, and this study suggests that they might be linked. Going forward, it will be of high relevance to investigate the mechanistic details underlying these phenotypes.

Previous studies have revealed that there are distinct fission signatures that are key determinants of the decision of mitochondria to proliferate or degrade (Kleele et al., 2021). Peripheral fission events, which occur near to the tips of mitochondria, are preceded by a decrease in membrane potential and proton motive force. We found that the elongated mitochondria from patient fibroblasts have a hyperpolarized membrane potential, which could result from inefficient function of the F_o_F_1_-ATPase. Under certain conditions, the F_o_F_1_-ATPase can pump protons out of the mitochondrial matrix into the intermembrane space in a reverse-mode action, causing ΔΨ_m_ hyperpolarization. Alternatively, hyperpolarization might be caused by complex I inhibition or voltage-dependent anion channel (VDAC) opening, which can cause hyperpolarization independently of F_0_F_1_–ATPase reverse-mode action (Forkink et al., 2014). Upregulation of glycolysis in these patient fibroblasts could result in subsequent glucose depletion locally within the cells, which could in turn cause ΔΨ_m_ hyperpolarization. Carbon-tracing could help reveal whether all products of glycolysis are efficiently shuttled into the TCA cycle or whether intermediates are lost in the process. Investigating the function of uncoupling proteins, such as UCP2 and UCP3, might help to elucidate whether the decreased efficiency is a byproduct or cause of mitochondrial dysfunction, such as increased ΔΨ_m_, in these cells.

Our finding that cells with DRP1 mutations have alterations in cristae morphology but apparent maintenance of overall ATP production and mitochondrial membrane hyperpolarization raises the interesting possibility that DRP1 mutant cells have evolved specialized mechanisms to cope with electron leakage and damaging ROS. A landmark study from Spinelli and collaborators demonstrated the ability of CII to work in reverse in some tissues with the intrinsic capacity to use fumarate as a terminal electron acceptor (Spinelli et al., 2021). It is plausible that CII reversal has evolved as a mechanism to maintain mitochondrial function in cells with alterations in fission caused by DRP1 mutations. A comprehensive analysis of the functionality of the ETC complexes would be needed to elucidate the connection between fission stalling events and mitochondrial hyperpolarization.

The metabolic profile of the mutant fibroblasts examined using HPLC-MS/MS suggest that abnormal mitochondrial fission results in impaired methionine cycle and synthesis of pyrimidine nucleotides. Dihydroorotate dehydrogenase (DHODH), located in the inner membrane of the mitochondria between CII and CIII, is an essential component of the *de novo* biosynthesis of pyrimidine (Boukalova et al., 2020). DHODH catalyzes the conversion of dihydroorotate into orotate in a redox reaction with ubiquinone (CoQ) converting it into ubiquinol (CoQH_2_), a substrate of respiratory complex III. A previous study (Miret-Casals et al., 2018), which aimed to identify novel mitochondrial fusion activators, reported that the depletion of pyrimidine pools induced by DHODH inhibition upregulates MFN1 and MFN2 and promotes mitochondrial elongation. Our study suggests yet another link between mitochondrial morphology and pyrimidine biosynthesis. Whether mitochondrial fission inhibition directly or indirectly affects pyrimidine synthesis presents an important area for future work.

The existence of two mechanistically and functionally distinct types of fission at the mitochondria and the peroxisomes have not been clearly elucidated. In our study, we demonstrate that patient fibroblasts with DRP1 mutations also have elongated peroxisomal morphology. Previous studies in mammalian cell culture revealed that peroxisome membrane elongation precedes membrane constriction and final membrane fission (Delille et al., 2010; Schrader et al., 2016). Whether peroxisomes have a specialized fusion machinery is still under debate. However, we speculate that under particular metabolic conditions, peroxisomes, particularly those in contact with mitochondria, might be able to fuse to respond to cellular metabolic needs. Elongation of peroxisomes in various human disorders has been associated with alterations in fatty acid oxidation (Waterham et al., 2016). However, in these cells, peroxisomal function cannot be fully dissociated from changes in mitochondrial morphology.

Our results suggest that MFN2 silencing could serve as a useful tool to discern the contribution of peroxisomal elongation independently of the mitochondrial network. However, some key technical limitations need to be overcome. Although knocking down MFN2 decreases the hyperfusion of the mitochondria in mutant cells, the mitochondrial dynamics would still be impaired. Another important variable is that MFN2 downregulation also has an effect in control cells, altering their mitochondrial dynamics. These results suggest that a fine-tuned balance needs to be achieved to ensure any metabolic defects caused by altered peroxisomal morphology can be uncoupled from those of mitochondrial hyperfusion. To investigate this in depth, recently published small-molecule inhibitors and activators of MFN2 (Zacharioudakis et al., 2022) could be used to explore the contribution of peroxisomal alteration to the metabolic defects reported in EMPF1 patients and patient-derived cells. These small molecules also provide the tools to determine the potential of pharmacological inhibition of mitochondrial fusion to restore tissue function in EMPF1.

Our model (Fig. 7D) suggests that the mechanistic and functional connection between mitochondrial and peroxisomal remodeling and metabolic homeostasis is dysregulated in the rare mitochondrial disease EMPF1. Although there is consensus on the mechanistic basis of the function of DRP1 in fission, our studies highlight new areas that require further investigation. To date, there are 40 EMPF1 cases reported in the literature with most mutations located in the GTPase or stalk domain (Batzir et al., 2019; Díez et al., 2017; Gerber et al., 2017; Hogarth et al., 2018; Keller et al., 2021; Ladds et al., 2018; Lhuissier et al., 2022; Longo et al., 2016; Nolden et al., 2022; Piccoli et al., 2021; Ryan et al., 2018; Schmid et al., 2019; Trujillano et al., 2017; Vanstone et al., 2016; Verrigni et al., 2019; Waterham et al., 2007; Wei and Qian, 2021; Whitley et al., 2018; Yoon et al., 2016; Zaha et al., 2016). There are clinical characteristics that are significantly different between the two domains, for example, abnormal electroencephalogram (∼20% of patients with GTPase domain mutations, and ∼92.9% of patients with mutations in the middle region), psychomotor delay (∼50% of patients with GTPase mutations, and ∼91.3% with middle domain mutations), and epilepsy (∼8.3% of patients with mutations in the GTPase domain, and ∼89.5% of patients with mutations in the middle domain) (Lhuissier et al., 2022; Liu et al., 2021). The clinical manifestations are more severe when patients have mutations in the middle domain. Understanding how mutations in different domains of DRP1 affect mitochondrial and/or peroxisomal fission and the effects of dysregulated fission for cellular homeostasis and function is imperative. In this context, we propose, that rare mitochondrial diseases can provide insight into the causal mechanisms underlying basic cellular processes, such as organelle fission.

The detailed elucidation of DRP1-centric mechanisms will reveal the connection between organelle dynamics and cell fate. Several questions remain. Does stalling of mitochondrial fission affect the association and/or dissociation of other proteins at the site of fission? Is peripheral fission also inhibited in patient fibroblasts? If that is the case, what are the consequences for mitophagy and mitochondrial DNA integrity? What are the effects of peroxisomal elongation on fatty acid metabolism and in ROS regulation? How is cell division and mitochondrial segregation affected by DRP1 mutations? How are cell fate transitions affected by DRP1 mutations? Answering these fundamental questions would provide insight into the mechanisms of mitochondrial fission under the lens of this rare disease caused by dysregulated mitochondrial and peroxisomal homeostasis and inform the field on more rational and specific therapeutic targets for these devastating neurodevelopmental disorders.

## MATERIALS AND METHODS

### Cell culture

Patient fibroblasts harboring the R403C mutation were obtained from Dr Eric Payne (Alberta Children's Hospital/The University of Calgary) (Ryan et al., 2018). Patient fibroblasts with the G32A mutation and an age-matched control with wild-type DRP1 were obtained from Dr Suzanne Hoppins (University of Washington, Seattle, WA) (Whitley et al., 2018). All fibroblast lines were obtained by skin biopsy and similar passages (±2) were used for all experiments. Human fibroblasts were thawed and plated directly in maintenance medium consisting of MEM (Gibco #11095098) with 10% FBS (Sigma-Aldrich #F2442) and non-essential amino acids (Gibco #11140050).

### Cell treatments

All treatments were added directly to fibroblasts in maintenance medium. The ETC decoupler CCCP (Sigma-Aldrich #C2759-250MG) was added to cells at a concentration of 1 µM. Cells were treated for 2 h prior to fixation and imaging. All stock solutions were prepared in DMSO.

### Plasmid transfection

Plasmid encoding mCherry-DRP1 (Addgene #49152) was transfected using FuGENE (Promega #E2311) as described in the manufacturer's protocol. Patient mutations were introduced to plasmids using the In-Fusion cloning kit (Takara Bio #638909) as described in the manufacturer's protocol. Cells were maintained until optimal transfection efficiency was reached before cells were imaged.

### siRNA transfection

siRNA knockdown against MFN2 (Thermo Fisher Scientific #s19260) or negative control scrambled was performed using Lipofectamine RNAiMAX transfection reagent (Thermo Fisher Scientific #13778150). Cells were fixed and stained 48 h after transfection.

### Immunofluorescence

For immunofluorescence, cells were fixed with 4% paraformaldehyde for 20 min and permeabilized in 1% Triton-X-100 for 5 min at room temperature. After blocking in 10% BSA, cells were treated with primary and secondary antibodies using standard methods. Cells were mounted in Fluoromount-G (Electron Microscopy Sciences #17984-25) prior to imaging. Information on antibodies can be found in Table S1.

### Western blot

Gel samples were prepared by mixing cell lysates with LDS sample buffer (Life Technologies, #NP0007) and 2-Mercaptoethanol (BioRad #1610710), then boiled at 95°C for 5 min. Samples were resolved on 4-20% Mini-PROTEAN TGX precast gels (BioRad #4561096) and transferred onto PVDF membrane (BioRad #1620177). Information on antibodies can be found in Table S1.

### Seahorse XF analysis

Fibroblasts were plated onto Seahorse XF96 V3 PS cell culture microplates 2 days before the assay at 50,000 cells per well. At 1 h prior to the assay, the medium was switched to XF DMEM containing 1 mM pyruvate, 2 mM glutamine and 10 mM glucose. For the Seahorse XF Mito Stress Test, the oxygen consumption rate (OCR) was measured sequentially after addition of 1.0 μM oligomycin, 0.5 μM FCCP and 0.5 μM rotenone plus antimycin A (all provided in Agilent #103015-100). Protein leak and coupling efficiency were calculated using the OCR. For the Seahorse XF glycolytic rate assay, the basal glycolytic rate was measured by extracellular acidification, and compensatory glycolysis was measured after addition of 0.5 μM rotenone plus antimycin A. Treatment with 50 mM 2-deoxyglucose (provided in Agilent #103344-100) was used as a negative control. For the Seahorse XF fuel flex test, glutamine dependency was measured by sequential addition of 3.0 μM BPTES followed by 4.0 μM Etomoxir plus 2.0 μM UK5099 (all provided in Agilent #103260-100). Fatty acid dependency was measured by sequential addition of 4.0 μM Etomoxir followed by 3.0 μM BPTES plus 2.0 μM UK5099. Glucose dependency was measured by sequential addition of 2.0 μM UK5099 followed by 3.0 μM BPTES plus 4.0 μM Etomoxir. For the Seahorse XF long-chain fatty acid oxidation stress test, 24 h prior to assay cells were switched to base medium containing 0.5 mM glucose, 1.0 mM glutamine, 0.5 mM XF L-carnitine, and 1.0% fetal bovine serum. At 1 h prior to the assay, the medium was switched to XF DMEM containing 2 mM glucose, 0.5 mM L-carnitine and palmitate-BSA (all provided in Agilent #103672-100). OCR was measured sequentially after addition of 4.0 μM etomoxir, 1.5 μM oligomycin, 2.0 μM FCCP and 0.5 μM rotenone plus antimycin A.

### O2K analysis

Oxygen consumption was measured with an O2K system (OROBOROS) using protocols 1 and 2 described in Ye and Hoppel (2013). Briefly, 2 ml of cell suspension (600,000 cells/ml) in Mir05 buffer was added to each chamber. Initial substrates were added (malate and pyruvate in protocol 1 and malate and palmitoylcarnitine in protocol 2) followed by addition of digitonin in increments (2 μg/ml) to permeabilize the cell membranes. The final concentration of substrates and inhibitors was composed of malate (2 mM), pyruvate (2.5 mM), adenosine diphosphate (ADP, 2.5 mM), glutamate (10 mM), succinate (10 mM), palmitoylcarnitine (10 μM), duroquinol (0.5 mM), etramethyl-p-phenylenediamine (TMPD, 0.5 μM), ascorbate (2 mM), carbonylcyanide-p-trifluoromethoxy-phenylhydrazone (FCCP, 0.5 μM increment), rotenone (75 nM), antimycin A (125 nM), and sodium azide (200 mM). For protocol 1, the non-mitochondrial (antimycin A-insensitive) rate was subtracted. For protocol 2, the Rot-insensitive rate was subtracted. For DHQ, the antimycin A was subtracted.

### RNA extraction and cDNA synthesis

Cells were washed with PBS and scraped into 1000 μl Trizol reagent (Invitrogen #15596018). 200 μl of chloroform (Sigma-Aldrich #C2432) was added and the samples were incubated at room temperature for 5 min. The samples were centrifuged at 12,000 ***g*** and the aqueous phase was collected. 500 μl of isopropanol was added to precipitate RNA, and the sample was incubated for 25 min at room temperature, followed by centrifugation at 12,000 ***g*** for 10 min at 4°C. The RNA pellet was washed with 75% ethanol, semi-dried, and resuspended in 30 μl of DEPC-treated water. After quantification and adjusting the volume of all the samples to 1 μg/μl, the samples were treated with DNase (New England Biolabs #M0303). 10 μl of this volume was used to generate cDNA using the manufacturer's protocol (Thermo Fisher Scientific, #4368814).

### Quantitative RT-PCR

1 μg of cDNA sample was used to run RT-qPCR for the primers mentioned in Table S1. QuantStudio 3 Real-Time PCR machine, SYBR green master mix (Thermo Fisher Scientific, #4364346), and manufacturer's instructions were used to set up the assay.

### Image acquisition

Super-resolution images were acquired using a Nikon SIM microscope equipped with a 1.49 NA 100× oil objective and an Andor DU-897 EMCCD camera. For SIM, all images used a step size of 0.12 μm for a total of 1.92 μm. Live-imaging experiments were acquired on a Nikon Eclipse Ti-E spinning disk confocal microscope equipped with a 1.40 NA 60× Oil objective or Apo TIRF 1.49 NA 100× objective and an Andor DU-897 EMCCD camera. For 100×, all images used a step size of 0.2 μm for a total of 1.8 μm, whereas the 60× used a step size of 0.3 μm for a total of 2.1 μm. Image processing and quantification for live imaging was performed using Fiji.

Quantification of mitochondrial morphology was performed in NIS-Elements (Nikon). Briefly, we segmented mitochondria in 3D and performed skeletonization of the resulting 3D mask. Skeleton major axis length, volume and surface area measurements were exported into GraphPad Prism v9.

### Sample preparation for TEM analysis

Human fibroblasts were immediately washed in ice-cold saline. Samples were processed as previously (de Assumpcão Pereira-da-Silva and Ferri, 2017; Lam et al., 2021). Cells were fixed in 2.5% glutaraldehyde in sodium cacodylate buffer for 1 h at 37°C, embedded in 2% agarose, postfixed in buffered 1% osmium tetroxide, stained in 2% uranyl acetate, and dehydrated with an ethanol graded series. After embedding in EMbed-812 resin, 80 nm sections were cut on an ultramicrotome and stained with 2% uranyl acetate and lead citrate. Images were acquired on a JEOL JEM-1230 transmission electron microscope, operating at 120 kV. Random images of human fibroblasts were obtained.

### SBF-SEM processing

Human fibroblasts were fixed in 2% glutaraldehyde in 0.1 M cacodylate buffer and processed using a heavy metal protocol adapted from a previously published protocol (Courson et al., 2021; Mustafi et al., 2014). Human fibroblasts were immersed in 3% potassium ferrocyanide and 2% osmium tetroxide (1 h at 4°C), followed by filtering in 0.1% thiocarbohydrazide (20 min), 2% osmium tetroxide (30 min), and left overnight in 1% uranyl acetate at 4°C (several de-ionized H_2_O washes were performed between each step). The next day samples were immersed in a 0.6% lead aspartate solution (30 min at 60°C and dehydrated in graded acetone (as described for TEM). Human fibroblasts were impregnated with epoxy Taab 812 hard resin, embedded in fresh resin, and polymerized at 60°C for 36–48 h. After polymerization, the block was sectioned for TEM to identify the area of interest, then trimmed to 0.5 mm×0.5 mm and glued to an aluminum pin. The pin was placed into an FEI/Thermo Scientific Volumescope 2 scanning electron microscope (SEM).

### TEM and SBF-SEM analysis

Measurements of mitochondrial area, circularity, and length were performed using the multi-measure region of interest (ROI) tool in ImageJ (Lam et al., 2021; Parra et al., 2014). To measure cristae morphology, we used ROIs in ImageJ to determine the cristae area, circulatory index, number, volume and cristae score. The NIH ImageJ software was used to manually trace and analyze all mitochondria or cristae using the freehand told in the ImageJ application (Lam et al., 2021; Parra et al., 2014).

### Mitochondrial event localizer

The MEL algorithm processes a fluorescence microscopy timelapse sequence of *z*-stack images of MitoTracker-labeled mitochondria and produces event counts per frame and 3D locations indicating where mitochondrial events are likely to have occurred at each time step (Theart et al., 2020). These locations can subsequently be superimposed on the *z*-stacks to indicate the different mitochondrial events. The algorithm was organized into two consecutive steps, namely the image pre-processing step, which normalizes and prepares the timelapse frames, and the automatic image analysis step, which calculates the location of the mitochondrial events based on the normalized frames. MEL also allows each event to be subsequently validated and removed from the visualization and event counts. The code is available for download at https://github.com/rensutheart/MEL-Fiji-Plugin.

### Metabolomics analysis

Control and patient fibroblasts were collected rinsed with ice-cold sterile 0.9% NaCl and flash-freeze in liquid nitrogen. For metabolite extraction, cells were resuspended in 225 μl of cold 80% HPLC-grade methanol/20% HPLC grade water per 10^6^ cells. After resuspension, cells were flash-frozen in liquid nitrogen and thawed rapidly in a 37°C water bath three times. Next, debris was removed by centrifugation at 16,000 ***g*** in a tabletop microcentrifuge at 4°C for 15 min. Metabolite-containing supernatant was transferred to a new tube, dried, and resuspended in 50% acetonitrile while the pellet was used for protein quantification. Samples were analyzed by ultra-high-performance liquid chromatography and high-resolution mass spectrometry and tandem mass spectrometry (UHPLC-MS/MS). Specifically, the system consisted of a Thermo Q-Exactive in line with an electrospray source and an Ultimate3000 (Thermo) series HPLC consisting of a binary pump, degasser, and auto-sampler outfitted with an Xbridge Amide column [Waters; dimensions of 4.6 mm×100 mm and a 3.5 μm particle size]. Mobile phase A contained 95% (v/v) water, 5% (v/v) acetonitrile, 10 mM ammonium hydroxide, 10 mM ammonium acetate, pH 9.0; mobile phase B was 100% acetonitrile. The gradient was as follows: 0 min, 15% A; 2.5 min, 30% A; 7 min, 43% A; 16 min, 62% A; 16.1–18 min, 75% A; 18–25 min, 15% A with a flow rate of 400 μl/min. The capillary of the ESI source was set to 275°C, with sheath gas at 45 arbitrary units, auxiliary gas at 5 arbitrary units, and the spray voltage at 4.0 kV. In positive–negative polarity switching mode, an *m*/*z* scan range from 70 to 850 was chosen, and MS1 data was collected at a resolution of 70,000. The automatic gain control target was set at 10^6^ and the maximum injection time was 200 ms. The top five precursor ions were subsequently fragmented, in a data-dependent manner, using the higher energy collisional dissociation cell set to 30% normalized collision energy in MS2 at a resolution power of 17,500. Data acquisition and analysis were carried out by Xcalibur 4.1 software and Tracefinder 4.1 software, respectively (both from Thermo Fisher Scientific). The peak area for each detected metabolite was normalized by the total ion current which was determined by the integration of all recorded peaks within the acquisition window. Five biological replicates per genotype were analyzed.

Normalized data was uploaded to MetaboAnalyst (https://www.metaboanalyst.ca/home.xhtml) for analysis. Samples were normalized to control, and a one-way analysis of variance (ANOVA) was performed to compare between the groups. Fisher's least significant difference method (Fisher's LSD) was performed as a post-HOC comparison. Enrichment and pathway analysis was also performed using this platform.

### Statistical analysis

All experiments were performed with a minimum of three biological replicates. Statistical significance was determined by one-way or repeated measures one-way ANOVA as appropriate for each experiment. Significance was assessed using Fisher's protected least significance difference test. GraphPad Prism v9 and Statplus software package were used for all statistical analysis (SAS Institute: Cary, NC, USA) and data visualization. Error bars in all bar graphs represent s.e.m. unless otherwise noted in the figure. No outliers were removed from the analyses. For all statistical analyses, a significant difference was accepted when *P*<0.05.

## Supplementary Material

10.1242/joces.260370_sup1 Supplementary information
