# Supplementary material for: DRP1 mutations associated with EMPF1 encephalopathy alter mitochondrial membrane potential and metabolic programs
Source: J Cell Sci. 2023 Feb 10;136(3):jcs260370. doi: 10.1242/jcs.260370 (PMC10657212; doi:10.1242/jcs.260370)
Supplement: Supplementary information [file joces-136-260370-s1.pdf]

Supplementary Figure 1

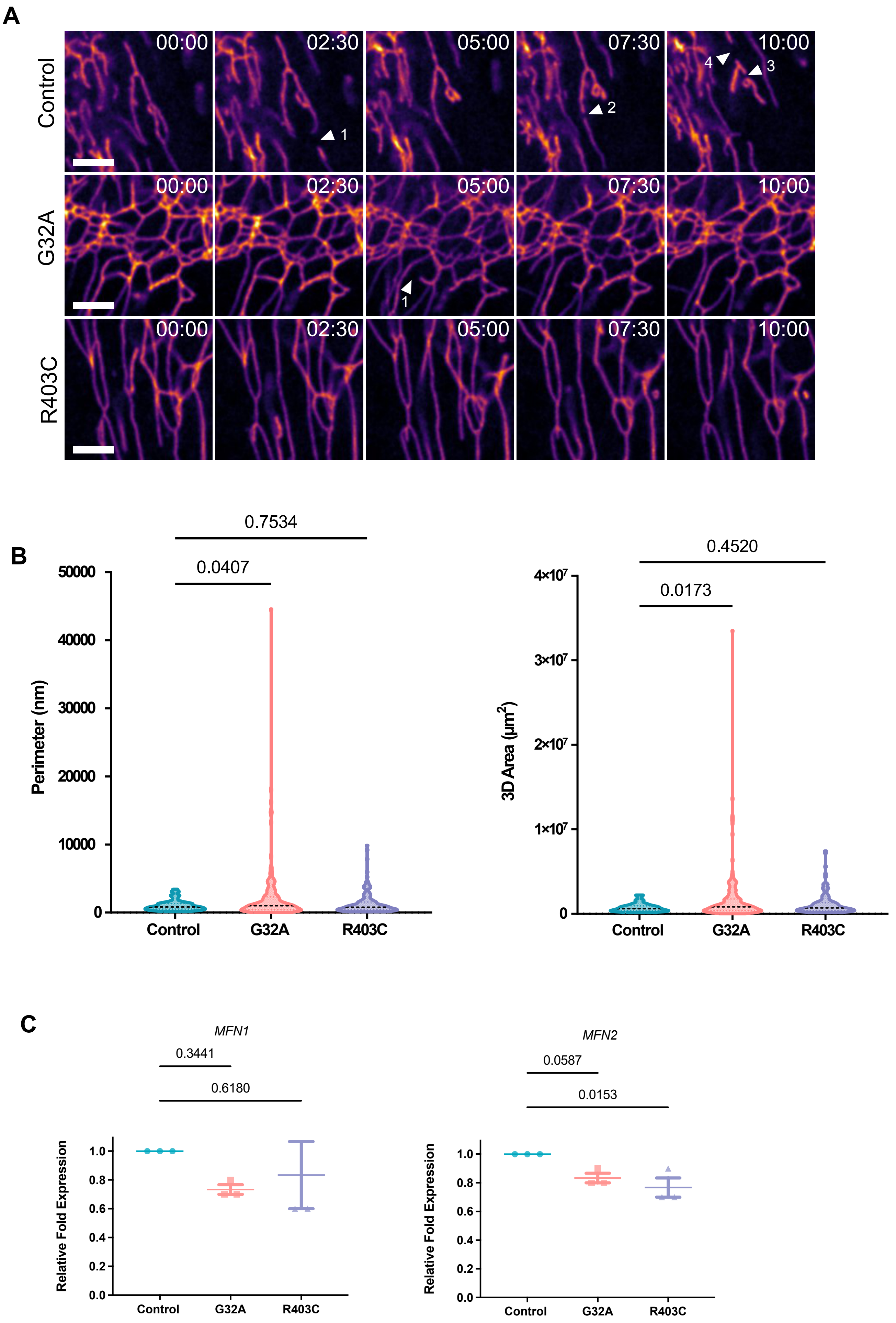

Journal of Cell Science • Supplementary information

Fig. S1. DRP1 patient fibroblasts rarely undergo mitochondrial fission events. (A) Live imaging of mitochondria in patient fibroblasts using MitoTracker (n=3, 5 cells per replicate). Cells were imaged every 15 sec for 30 min. Arrows indicate points of fission observed. Scale bar: 1 µm. (B) Quantification of mitochondrial perimeter and 3D area from SBF-SEM. (C) qRT-PCR analysis of *MFN1* and *MFN2* gene expression in fibroblasts relative to control and normalized to two housekeeping genes (*GPI* and *GAPDH)*. Quantifications are shown for three independent biological replicates.

Journal of Cell Science • Supplementary information

Supplementary Figure 2

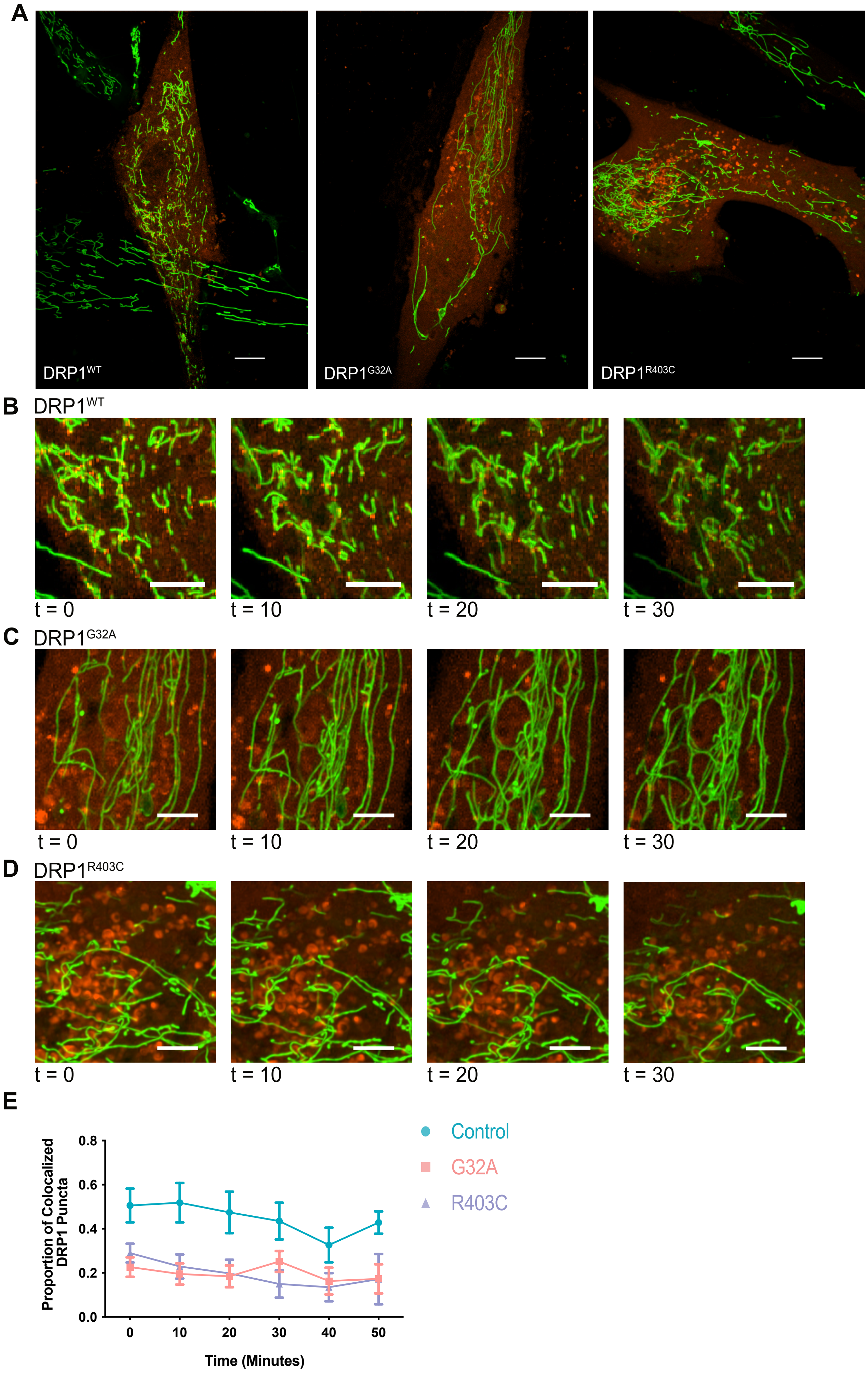

Journal of Cell Science • Supplementary information

Fig. S2. mCherry-DRP1 localization dynamics in live cells. (A) Representative max-intensity projections at t=0 of live imaging of control fibroblasts transiently transfected with mCherry-DRP1 (WT, G32A, or R403C) using MitoTracker Green (n=3, 5 cells er replicate). Cells were imaged every 10 min for 60 min. Scale bar: 10 µm. (B) Zoom of mCherry- RP1-WT mitochondrial localization over time. Scale bar: 5 µm. (C) Zoom of mCherry-DRP1- G32A mitochondrial localization over time. Scale bar: 5 µm. (D) Zoom of mCherry-DRP1-R403C itochondrial localization over time. Scale bar: 5 µm. (E) Quantification of proportion of mCherry- DRP1 localized to the mitochondria over time.

Supplementary Figure 3

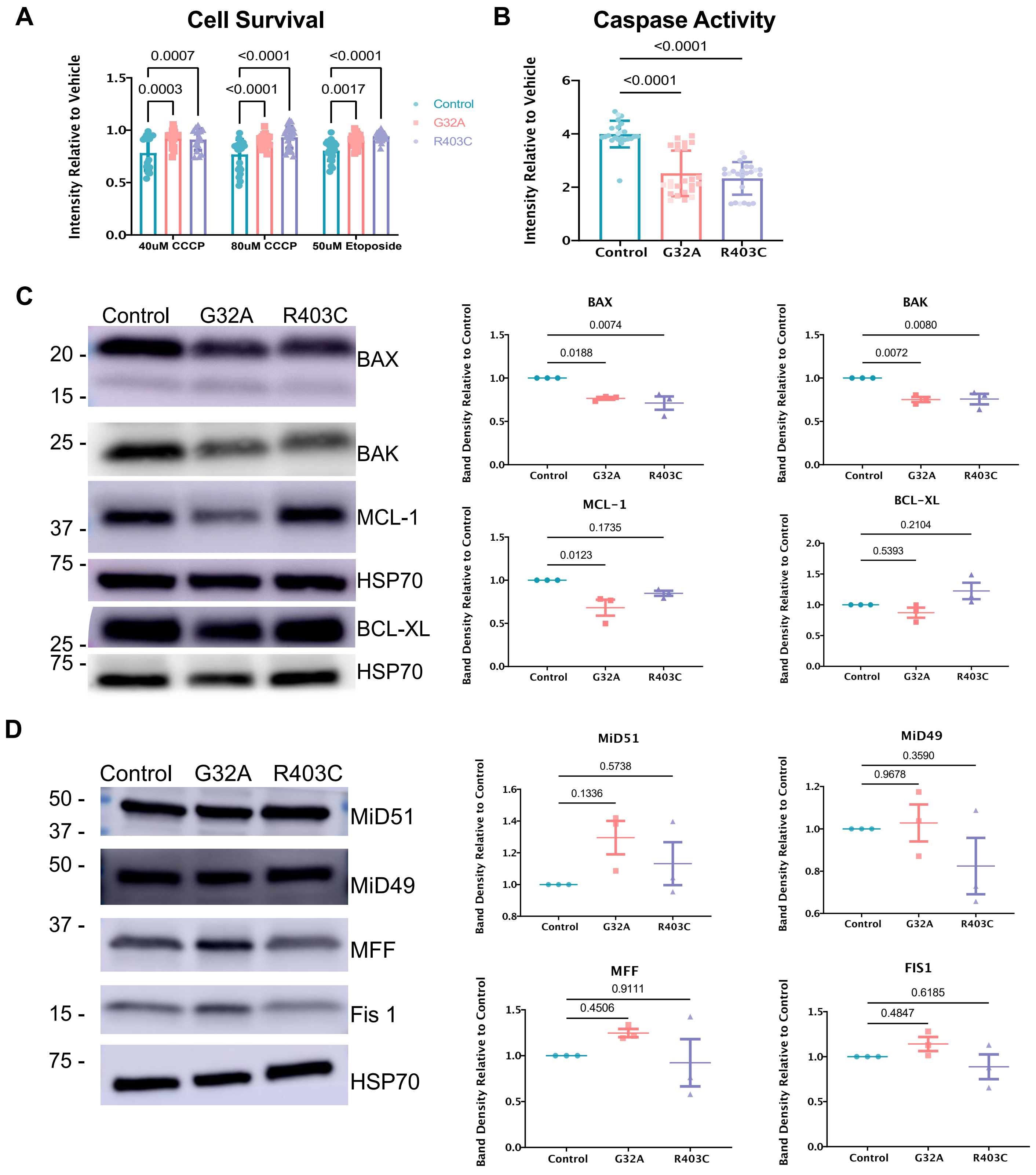

Journal of Cell Science • Supplementary information

Fig. S3. DRP1 patient fibroblasts express equal levels of adaptor proteins but downregulate pro-apoptotic proteins. (A) Cell titer blue assay with results relative to vehicle treated cells of same genotype. Analyzed using two-way ANOVA with Dunnett's multiple comparison test. (B) Caspase-Glo 3/7 assay with results relative to vehicle treated cells of same genotype. Analyzed using two-way ANOVA with Dunnett's multiple comparison test. (C) Western blot of total protein lysate isolated from patient fibroblasts and probed for pro and anti-apoptotic proteins. (D) Western blot of total protein lysate isolated from patient fibroblasts and probed for mitochondrial fission adaptor proteins. All quantified relative control and normalized to loading control (HSP70).

Journal of Cell Science • Supplementary information

Supplementary Figure 4

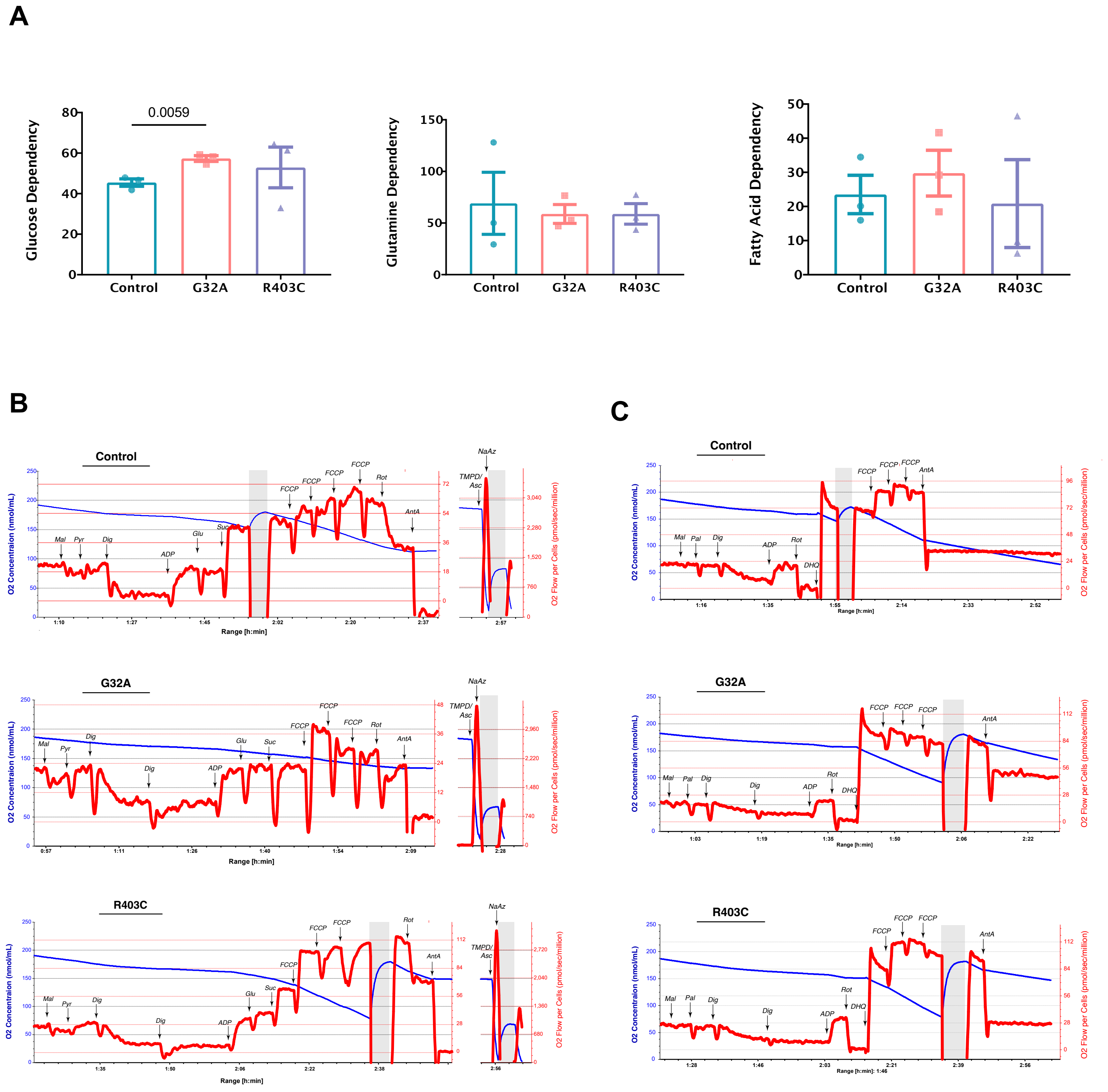

Journal of Cell Science • Supplementary information

Fig. S4. DRP1 patient fibroblasts rely more on glucose for fuel and downregulate expression of the mitochondrial pyruvate carriers. (A) Glucose, glutamine, and fatty acid dependency of the electron transport chain measured using Seahorse Fuel Flex Assay. Glucose dependency measured after application of UK5099, glutamine dependency measured after application of BPTES, and fatty acid dependency measure after application of Etomoxir. (B) Representative O2 concentration traces from protocol 1. (C) Representative O2 concentration traces from protocol 2.

Journal of Cell Science • Supplementary information

Supplementary Figure 5

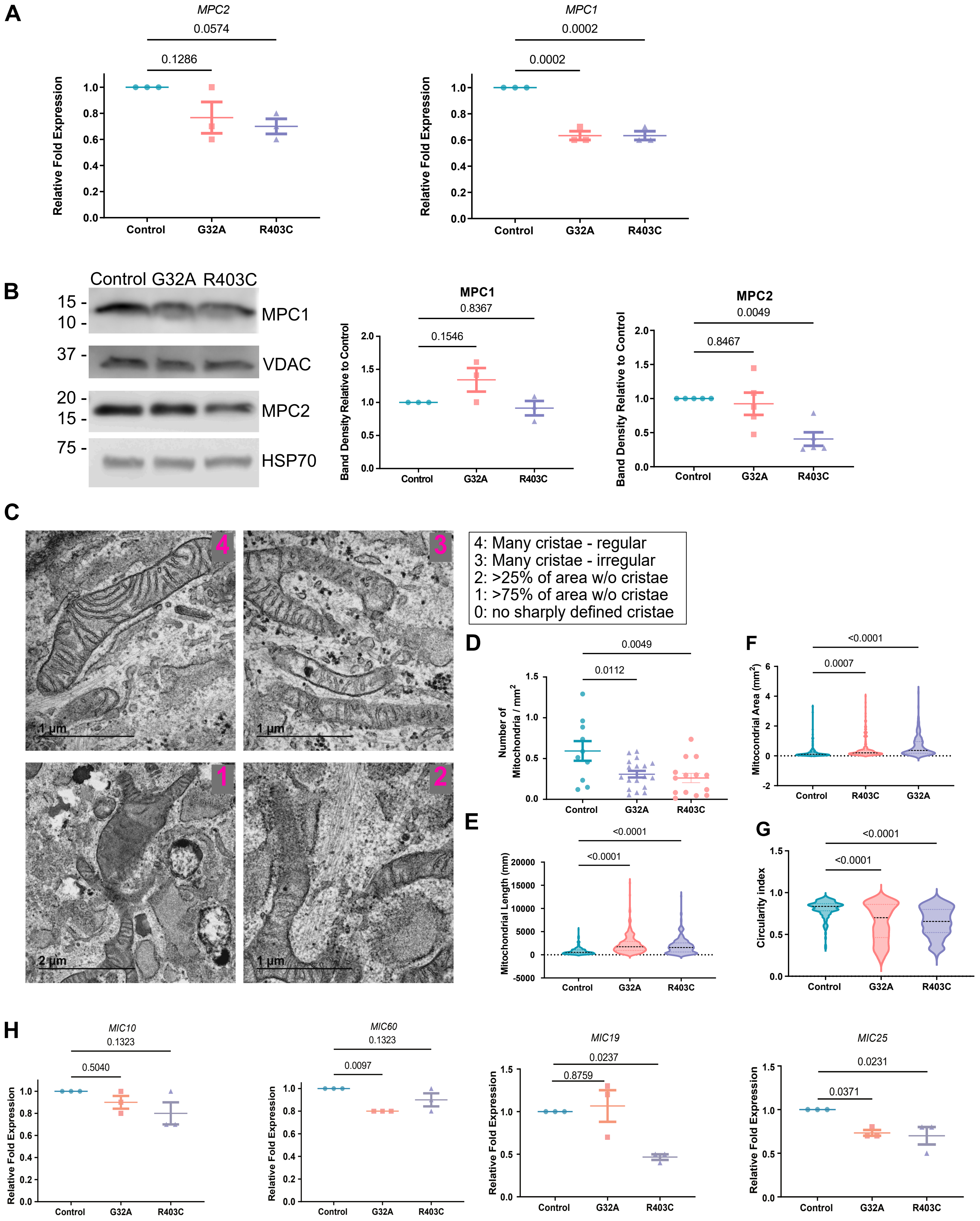

Journal of Cell Science • Supplementary information

Fig. S5. TEM cristae scoring examples and gene expression of MICOS complex components. (A) qRT-PCR analysis of *MPC1* and *MPC2* gene expression in fibroblasts relative to control and normalized to two housekeeping genes (*GPI* and *GAPDH)*. Quantifications are shown for three independent biological replicates. (B) Western blot of total protein lysate isolated from patient fibroblasts and probed for mitochondrial pyruvate carriers. Band density is normalized to loading control (HSP70) and relative to control cells. Representative image of three independent biological replicates. (C) Representative TEM images of mitochondria with cristae fitting the given cristae score. (D) Quantification of TEM images for mitochondria number, (E) mitochondrial length, (F) mitochondrial area, and (G) mitochondrial circularity index. (H) qRT-PCR analysis of MICOS components gene expression in fibroblasts relative to control and normalized to two housekeeping genes (*GPI* and *GAPDH*).

Journal of Cell Science • Supplementary information

Supplementary Figure 6

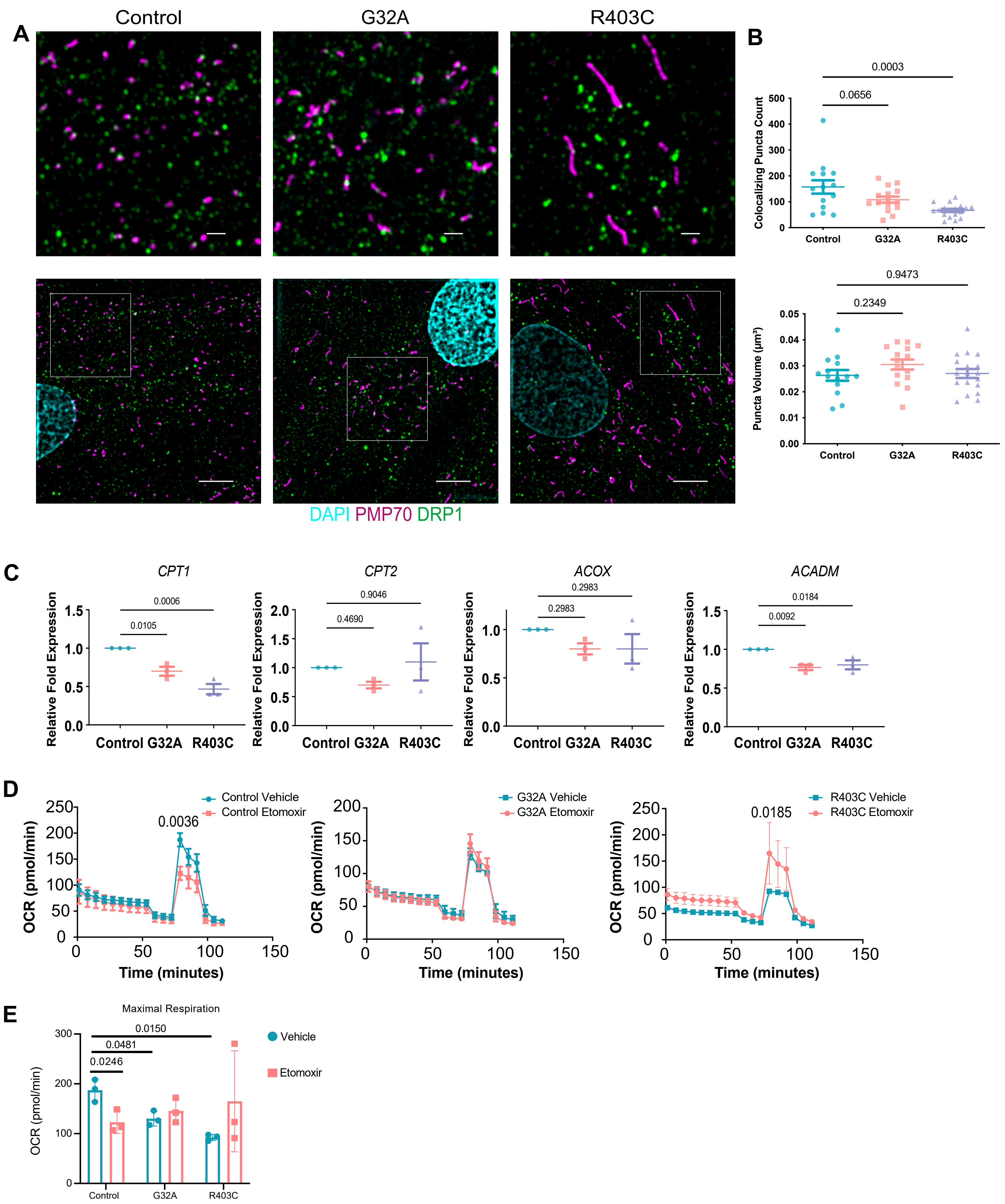

Journal of Cell Science • Supplementary information

Fig. S6. Fatty acid metabolism may be altered in patient fibroblasts.
(A) Representative images of peroxisomes and DRP1 in patient-derived fibroblasts using structured illumination microscopy (n=3, 7 cells per replicate). Top image is a zoom of white box from bottom image. Scale bar: 1 µm (top), 5 µm (bottom) (B) Quantification of DRP1 puncta that overlap with peroxisomes and volume of DRP1 puncta. Analyzed using one-way ANOVA followed by Dunnett's multiple comparison test. (C) qRT-PCR analysis of metabolic enzymes gene expression in fibroblasts relative to control and normalized to two housekeeping genes (*GPI* and *GAPDH)*. Quantifications are shown for three independent biological replicates. (D) Oxygen consumption rate in patient fibroblasts as measured using Seahorse Analyzer XFe96 (n=3, 20 wells per replicate). Etomoxir applied after 20 min, oligomycin applied at 40 min, FCCP applied t min, and rotenone/Antimycin A applied at 80 min. Analyzed using two-way ANOVA followed y Šídák's multiple comparisons test. (E) OCR at maximal respiration after FCCP treatment. Analyzed using one-way ANOVA followed by Dunnett's multiple comparison test.

Supplementary Figure 7

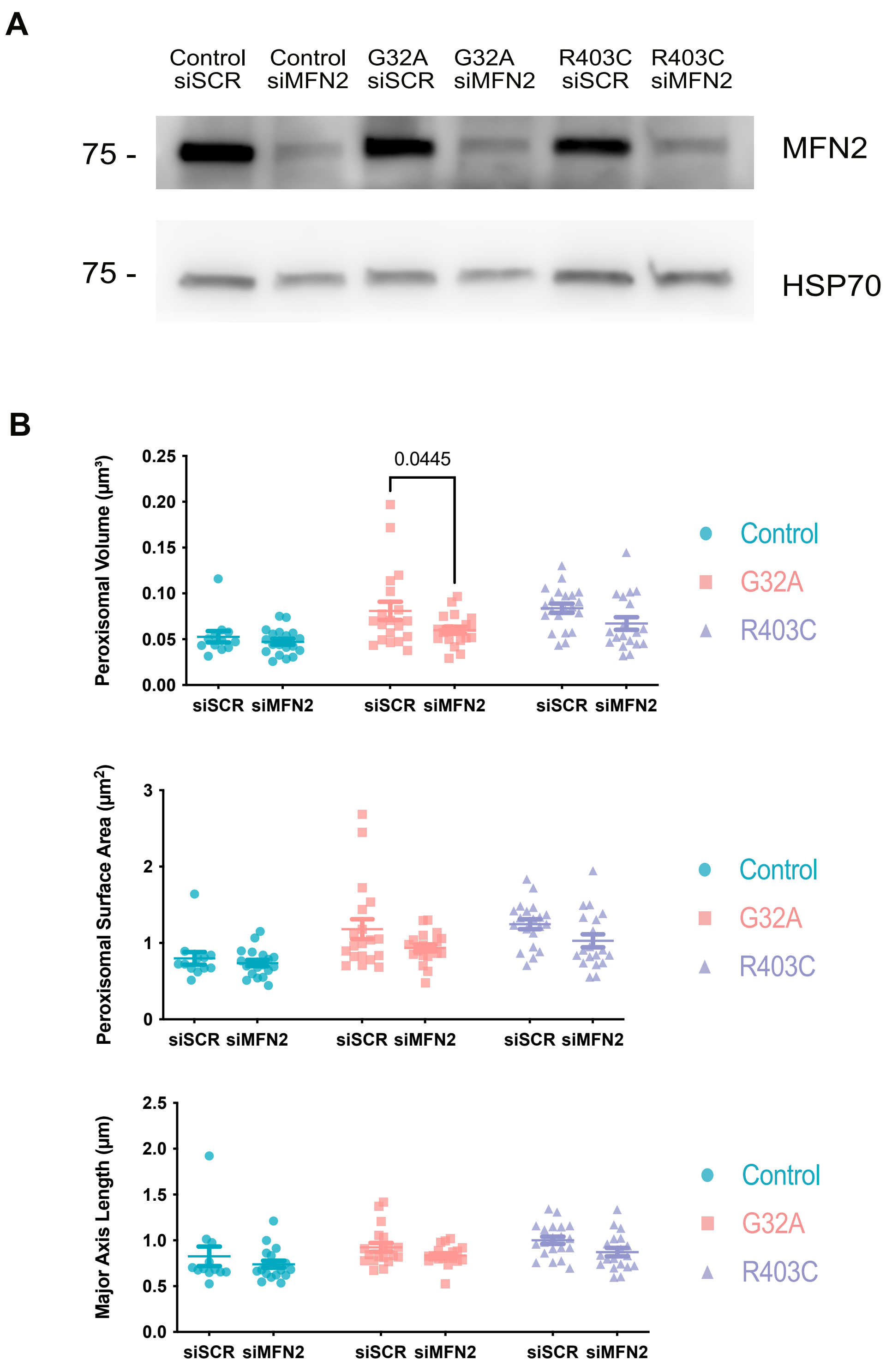

Fig. S7. Peroxisomal morphology is mostly unchanged after MFN2 knockdown. (A) Western blot of total protein lysate isolated from patient fibroblasts treated with non-specific siSCR or siMFN2. Representative image of three independent biological replicates. (B) Quantification of peroxisomal volume, surface area, and major axis length. Analyzed using one-way ANOVA followed by Dunnett's multiple comparison test.

Journal of Cell Science • Supplementary information

Table S1. KEY RESOURCES

REAGENT or RESOURCE	SOURCE	IDENTIFIER
Antibodies		
Mouse anti-mitochondria [113-1]	Abcam	Cat#Ab92824<br>RRID:AB_10562769
Rabbit anti-Tom20	Cell Signaling Technologies	Cat# 42406S<br>RRID:AB_2687663
Rabbit anti-DRP-1	Cell Signaling Technologies	Cat#8570S; RRID:AB_10950498
Rabbit anti-pDRP-1 S616	Cell Signaling Technologies	Cat#3455S; RRID:AB_2085352
Rabbit anti-OPA1	Cell Signaling Technologies	Cat#67589S; RRID:AB_2799728
Mouse anti-Mitofusin 1	Abcam	Cat# ab57602, RRID:AB_2142624
Mouse anti-Mitofusin 2	Abcam	Cat# ab56889, RRID:AB_2142629
Mouse anti-Hsp70	Thermo Fisher Scientific	Cat# MA3-006, RRID:AB_325454
Rabbit anti-MPC1	Atlas Antibodies	Cat# HPA045119, RRID:AB_10960421
Rabbit anti-MPC2	Proteintech	Cat# 20049-1-AP, RRID:AB_10665371
Mouse anti-PMP70	Thermo Fisher Scientific	MA5-31368, RRID:AB_2787005
Rabbit anti-Bax	Cell Signaling Technologies	Cat# 5023, RRID:AB_10557411
Rabbit anti-Bak	Cell Signaling Technologies	Cat# 12105, RRID:AB_2716685
Rabbit anti-BCL-XL	Cell Signaling Technologies	Cat# 2764, RRID:AB_2228008
Rabbit anti-MCL-1	Cell Signaling Technologies	Cat#94296S; RRID:AB_2722740
Alexa Fluor-488 anti-rabbit IgG	Thermo Fisher Scientific	Cat# A-21206, RRID:AB_2535792
Alexa Fluor-488 anti-mouse IgG	Thermo Fisher Scientific	Cat# A-21202, RRID:AB_141607

Journal of Cell Science • Supplementary information

Alexa Fluor-546 anti-rabbit IgG	Thermo Fisher Scientific	Cat# A10040, RRID:AB_2534016
Alexa Fluor-546 anti-mouse IgG	Thermo Fisher Scientific	Cat# A10036, RRID:AB_2534012
Chemicals, Peptides, and Recombinant Proteins		
CCCP ETC decoupler	Sigma-Aldrich	Cat#C2759-250MG
FuGENE transfection reagent	Promega	Cat#E2311
TMRE	Sigma Aldrich	Cat# 7917-25MG
Critical Commercial Assays		
In Fusion cloning kit	Takara Bio	Cat# 638909
Seahorse XF Cell Mito Stress Test Kit	Agilent	Cat#103015-100
Seahorse XF Glycolytic Rate Assay	Agilent	Cat#103344-100
Seahorse XF Mito Fuel Flex Test Kit	Agilent	Cat#103260-100
Seahorse XF Long Chain Fatty Acid Oxidation Stress Test	Agilent	Cat#103672-100
Recombinant DNA		
Plasmid encoding mCherry-DRP1	Addgene	Cat#49152; RRID:Addgene_49152
Primers		
MFN1 Forward: GAGGTGCTATCTCGGAGACAC<br>MFN1 Reverse: GCCAATCCCACTAGGGAGAAC	Integrated DNA Technologies	N/A
MFN2 Forward: CACATGGAGCGTTGTACCAG<br>MFN2 Reverse: TTGAGCACCTCCTTAGCAGAC	Integrated DNA Technologies	N/A
MPC1 Forward: ATTTGCCTACAAGGTACAGCC<br>MPC1 Reverse: AGTCATCTCGTGTTTGATAAGCC	Integrated DNA Technologies	N/A
MPC2 Forward: TACCACCGGCTCCTCGATAAA<br>MPC2 Reverse: TATCAGCCAATCCAGCACACA	Integrated DNA Technologies	N/A
MIC10 Forward: GATGCGGTCGTGAAGATAGGT<br>MIC10 Reverse: CCAGAACCGAAGGCTAATGG	Integrated DNA Technologies	N/A
MIC60 Forward: CGGGCCTGTCAGTTATCGG<br>MIC60 Reverse: CAATGGACGGAGGACAAACTT	Integrated DNA Technologies	N/A

Journal of Cell Science • Supplementary information

MIC19 Forward: GAGGCGGACGAGAATGAGAAC<br><br>MIC19 Reverse: ACCAGAATACCGCTGAGACTTC	Integrated DNA Technologies	N/A
MIC25 Forward: ACCAAGCACTCCAAGGCATC<br><br>MIC25 Reverse: GTGTCACGGCGTCTTAGCTC	Integrated DNA Technologies	N/A
CPT1 Forward: GCGCCCCTTGTTGGATGAT<br><br>CPT1 Reverse: CCACCATGACTTGAGCACCAG	Integrated DNA Technologies	N/A
CPT2 Forward: CTGGAGCCAGAAGTGTTCCAC<br><br>CPT2 Reverse: AGGCACAAAGCGTATGAGTCT	Integrated DNA Technologies	N/A
ACOX Forward: GCACCCCGACATAGAGAGC<br><br>ACOX Reverse: CTGCGGAGTGCAGTGTTCT	Integrated DNA Technologies	N/A
ACADM Forward: TGGATAACCAACGGAGGAAAAG<br><br>ACADM Reverse: CTGGGGTATCTGCTTCCACA	Integrated DNA Technologies	N/A
Software and Algorithms		
Fiji	Schindelin et al., 2012	https://imagej.net/Fiji
GraphPad Prism v8.1.2	GraphPad	https://www.graphpad.com/scientific-software/prism/
NIS-Elements	Nikon	
Affinity Designer	Affinity	https://affinity.serif.com/en-us/designer/
Image Studio Lite	LI-COR	https://www.licor.com/bio/image-studio-lite/
Other		
Seahorse XF calibrant	Agilent	Cat#100840-000
Seahorse XF96 V3 PS cell culture microplates	Agilent	Cat#101085-004
Seahorse XFe96 Extracellular flux assay kits	Agilent	Cat#102601-100
Seahorse XFe96 Analyzer	Agilent	N/A
Seahorse XF DMEM medium pH 7.4	Agilent	Cat#103575-100
Seahorse XF 1.0 M glucose solution	Agilent	Cat#103577-100
Seahrose XF 100mM pyruvate solution	Agilent	Cat#103578-100
Seahorse XF 200 mM glutamine solution	Agilent	Cat#103579-100

Journal of Cell Science • Supplementary information
