# Supplementary material for: DRP1 mutations associated with EMPF1 encephalopathy alter mitochondrial membrane potential and metabolic programs
Source: J Cell Sci. 2023 Feb 10;136(3):jcs260370. doi: 10.1242/jcs.260370 (PMC10657212; doi:10.1242/jcs.260370)
Supplement: Supplementary file 1 [file joces-136-260370_review_history.pdf]

DRP1 mutations associated with EMPF1 encephalopathy alter mitochondrial membrane potential and metabolic programs

Gabriella L. Robertson, Stellan Riffle, Mira Patel, Caroline Bodnya, Andrea Marshall, Heather Beasley, Edgar Garza Lopez, Jianqiang Shao, Zer Vue, Antentor Hinton Jr., Maria S. Stoll, Sholto de Wet, Ram Prosad Chakrabart, Rensu P. Theart, Ben Loos, Navdeep Chandel, Jason A. Mears and Vivian Gama
DOI: 10.1242/jcs.260370

Editor: Jennifer Lippincott-Schwartz

Review timeline

Original submission:	27 June 2022
Editorial decision:	22 August 2022
First revision received:	13 December 2022
Accepted:	22 December 2022

Original submission

First decision letter

MS ID#: JOCES/2022/260370

MS TITLE: DRP1-mediated mitochondrial fission is essential to maintain cristae morphology and bioenergetics

AUTHORS: Gabriella L. Robertson, Stellan Riffle, Mira Patel, Andrea Marshall, Heather Beasley, Edgar Garza Lopez, Jianqiang Shao, Zer Vue, Maria S. Stoll, Sholto de Wet, Rensu P. Theart, Ben Loos, Antentor Hinton Jr., Jason A. Mears, and Vivian Gama
ARTICLE TYPE: Research Article

We have now reached a decision on the above manuscript.

To see the reviewers' reports and a copy of this decision letter, please go to: https://submit-jcs.biologists.organd click on the 'Manuscripts with Decisions' queue in the Author Area. (Corresponding author only has access to reviews.)

As you will see, the reviewers raise a number of substantial criticisms that prevent me from accepting the paper at this stage. They suggest, however, that a revised version might prove acceptable, if you can address their concerns. If you think that you can deal satisfactorily with the criticisms on revision, I would be pleased to see a revised manuscript. We would then return it to the reviewers. Please focus on points 3,4,6 and 7 from Reviewer #1 and address all the other points raised by Reviewers #2 and 3.

Please ensure that you clearly highlight all changes made in the revised manuscript. Please avoid using 'Tracked changes' in Word files as these are lost in PDF conversion.

I should be grateful if you would also provide a point-by-point response detailing how you have dealt with the points raised by the reviewers in the 'Response to Reviewers' box. Please attend to all of the reviewers' comments (except 1, 2 and 5 from reviewer #1). If you do not agree with any of their criticisms or suggestions please explain clearly why this is so.

© 2023. Published by The Company of Biologists under the terms of the Creative Commons Attribution License (https://creativecommons.org/licenses/by/4.0/).

Reviewer 1

Advance summary and potential significance to field

In this manuscript by Gama and colleagues, the authors utilize patient-derived fibroblasts to functionally characterize the mitochondrial, peroxisomal, and metabolic defects associated with two disease-associated heterozygous mutations in the gene encoding for the mitochondrial fission protein Drp1. The authors confirmed previous analysis of these Drp1 mutations (G32A and R403C), which have been shown to compromise Drp1 function in mitochondrial fission and act in a dominant-negative manner. The authors extend the analysis of Drp1 mutation, for example, to demonstrate that they affect protein stability of mitofusins, mildly affect cristae morphology, lead to resistance against apoptosis induction, and cause altered peroxisomal morphology. The authors also perform metabolic analysis of these patient-derived lines and find reduced mRNA expression of two beta-oxidation pathway genes and potential differential beta-oxidation utilization.

Comments for the author

The work is generally well performed and the imaging is of high quality. However, most of the observed findings are consistent with previously published characterization of these specific patient mutations and/or are phenotypes already associated with loss of Drp1 function. For example, several studies have done seahorse assays on Drp1 KD/KO cells and fatty acid oxidation utilization has also been evaluated (for example, King et al., JBC, 2021). The observations here (such as the reduction of mRNA levels of two beta-oxidation pathway genes), while somewhat interesting, are not followed up in depth. Thus, while there is no doubt that the findings of the manuscript are worthy of publication, the motivation for the study is not completely clear and there is not significant enough conceptual advance to recommend publication in JCS.

1. The examination of beta-oxidation capacity in cells defective of mitochondrial fission is potentially interesting, though underexplored. What does it mean that there is reduced mRNA expression (20-50% lower) of CPT1 or ACADM? Does this correspond to lower protein level? For example, in the authors' analysis, they mentioned that they found lower mRNA expression for MICOS subunits that was not observed to correspond to protein stability defects though this data was not shown. Is fatty acid uptake capability diminished or are beta-oxidation rates altered? The authors did a Seahorse assay on cells treated with etomoxir and found patient lines had different maximal ATP production, but the mechanistic basis for this is lacking. How do these defects relate to the patient disease phenotypes?

2. Are the alterations in metabolic capacity of patient lines rescued by simultaneous depletion of fusion machinery? The authors note in the discussion that this could be used as a tool to uncouple the metabolic defects of peroxisomal shape alteration from those of mitochondrial hyperfusion. The manuscript would be strengthened by evaluating this idea.

3. The section of the manuscript on mitochondrial respiratory activity was technically dense and made confusing by the use of non-standard acronyms (P + M Pal, Rot). The manuscript would be strengthened by a clearer explanation of the rationale for such thorough analysis and how such results informed follow up experiments. For example, what do the differences observed between R403C and the G32A mutation mean? How does this correspond to patient phenotypes?

4. The title of the paper is misleading as cristae morphology, while slightly altered, is not grossly affected in the Drp1 patient lines. Cristae are certainly affected by loss of Drp1, but whether the process of fission itself is required for their maintenance is not directly evaluated. Additionally, effects of Drp1 KD on cristae morphology have been previously reported (Ban-Ishihara et al., PNAS, 2013).

5. The authors make the point that mitochondrial morphology is corrected by MFN2 depletion while peroxisomal morphology is not, though images are not shown of the peroxisomes.

6. Hyperpolarization of mitochondria as assessed by TMRE staining should be normalized to a non-membrane potential dependent mitochondrial label such as mitotracker green.

© 2023. Published by The Company of Biologists under the terms of the Creative Commons Attribution License (https://creativecommons.org/licenses/by/4.0/).

7. Line 380 – the authors refer to siRNA of MFN2 as deletion. Also, no western blots are shown of MFN2 (or MFN1) in this knockdown.

Reviewer 2

Advance summary and potential significance to field

In their manuscript Robertson et al. perform detailed and high-quality characterization of the mitochondrial network and metabolism in fibroblasts derived from patients harboring de novo missense mutations in the DNM1L gene.
DNM1L encodes DRP1, a protein that is essential for executing both mitochondrial and peroxisomal fission by mediating outer membrane constriction. Both mutations studied (G32A and R403C) are heterozygous in patients and have been reported to exert a dominant-negative effect on mitochondrial fission when overexpressed on the background of wild-type DRP1. In keeping with previous studies, the authors observe a hyperfused mitochondrial network in both G32A and R403C patient cell lines by SIM, block-facing scanning EM and transmission EM. TEM image analysis reveals that the hyperfused mitochondria have fewer and more irregular cristae.
Additionally, SIM imaging shows a hyperfused peroxisomal network. The authors find a number of functional consequences of the mitochondrial hyperfusion phenotype: a delayed apoptotic response to stress, a decrease in the ETC coupling efficiency an increase in glycolysis rate and interestingly hyperpolarization of the inner mitochondrial membrane. Both mitochondrial and peroxisomal hyperfusion phenotypes may contribute to the decreased dependance of patient fibroblasts on fatty acid oxidation. Although mitochondrial fusion machinery proteins Mitofusin 1 and Mitofusin 2 are already downregulated in patient fibroblasts, further depletion of Mitofusin 2 by siRNA rescued the mitochondrial fusion phenotype.

Compared to previous publications characterizing dominant-negative DRP1 mutations this study provides an in-depth characterization of consequences of mitochondrial hyperfusion on cellular metabolism. Although patient-derived iPSC neurons would be more relevant to study EMPF1 pathology, the careful dissection provided here will provide a solid basis for future studies. Some revisions are necessary to ensure that the data reported justifies the conclusions drawn.

Comments for the author

Major points:

1) The authors provide a model (Figure 7), in which dominant-negative DRP1 mutants do not prevent recruitment of DRP1 to the mitochondrial surface, but instead result in stalled fission intermediates where DRP1 is present, yet unable to facilitate complete membrane fission. In Figures 2C and 2D the authors use immunofluorescence to show no difference in the number and volume of pS616-DRP1 mitochondrial puncta between control and patient-derived fibroblasts. (I assume there is an error in the figure legend here which should be addressed.) In the main text description of this figure (line 187-188) the authors state that 'in the patient fibroblasts this interaction appeared less transient'. It is not clear to me how this conclusion can be made from the fixed-cell immunofluorescence data provided. In order to support their claim, the authors should consider time-resolved imaging of co-localizing puncta in cells overexpressing DRP1 variants as in Figures 1F and 1G. Whitley et al., 2018 report that while overexpression of GFP-fusion constructs of both the G32A and R403C variants result in a mitochondrial hyperfused phenotype, overexpressed GFP-DRP1-G32A remained entirely cytosolic and was not recruited to mitochondrial puncta. These findings should be discussed in light of the proposed model.
2) The authors argue that the reduction of Mitofusin 1 and 2 levels are the result of protein destabilization associated with the lack of mitochondrial fission without checking transcript levels. To support their conclusion, it is necessary to check Mitofusin transcript levels by qPCR, as has been done for other mitochondrial proteins assessed in this study.

© 2023. Published by The Company of Biologists under the terms of the Creative Commons Attribution License (https://creativecommons.org/licenses/by/4.0/).

Minor points:

1) It is not clear whether control fibroblasts match characteristics of the patients from whom the G32A and R403C lines were derived. The authors should discuss the source for all three described cell lines in the Materials and Methods section.
2) To aid interpretation of Figure 3 as well as the main text description of O2K respiration measurements, it would be helpful for the authors to include representative traces for both protocols in a supplementary figure, indicating at what points values are taken for comparison in the main figure. Could the authors clarify why there are different trends in the Intact Cellular Respiration rate at the beginning of O2K Protocol 1 vs Protocol 2? The discussed elevated ICR rate observed for the R403C line in Protocol 2 before and after Malate addition does not seem to be reproduced in Protocol 1.
3) The mitochondrial hyperpolarization in both patient cell lines is interesting, however quantification of this phenomenon is necessary. Would it be possible to show quantification across all cells in the reported independent experiments?
4) Figures 7C and 7D are not referred to in the main text – I believe they should be referred to in place of Figure 7B on line 381.

Reviewer 3

Advance summary and potential significance to field

In Robertson et al., the authors aimed to determine the cellular consequences of two prior reported single nucleotide Drp1 variants that cause encephalopathy due to defective mitochondrial and peroxisomal fission (EMPF1) in patients, one in the GTPase domain (G32A) and one in the stalk domain (R403C). To accomplish this, the authors used a combination of live cell fluorescence and super-resolution microscopy on both patient-derived fibroblasts and control fibroblasts undergoing transient transfection, transmission electron microscopy to evaluate mitochondrial cristae structure, cellular metabolism measurements and both gene and protein expression quantification. In doing so, the authors perform a thorough characterization of the two EMPF1-associated variants and perform one of the most thorough metabolic analyses to date for pathological Drp1 variants. In fact the manuscripts presents many firsts in the field with respect to Drp1 pathological variants (first detailed report of how pathological variants of Drp1 impact cellular respiration, first use of super-resolution first investigation of Drp1 Ser616 phosphorylation in pathological variants first counting of fission and fusion events, and others. Further, the authors expand on the prior studies in which these variants were initially identified concluding that not only is mitochondrial fission disrupted, but so is the frequency of fission events, mitochondrial membrane potential, and cell response to apoptotic stimuli. Further, peroxisomal fission, and levels of peroxisomal proteins involved in fatty acid metabolism, are dysregulated.

Overall, the experiments in this study are extremely thorough and of high quality. In addition, the authors are cognizant of the limitations of the study.
The manuscript would be improved by more of a discussion on how their findings fit in the context of the growing number of pathological Drp1 variants that have been identified and characterized. Further, it is interesting that mutations in two different domains can lead to similar functional impairments, albeit with different clinical presentations and apparent mechanistic causes. Other mutations within the GTPase and stalk domain have also been characterized typically with vastly different constellations of patient symptoms and disease severity. Given this, it would have been interesting to have more insight into why the authors thought these differential effects might exist.

Comments for the author

Main comments
1. Insufficient details in many places
a. Throughout methods section, details on data collection are scant (e.g. z-slices for Fig 7).
b. Details on microscopy data analysis throughout are lacking in places. E.g. how mitochondrial volume, surface area, and major axis length are determinined with Nikon Elements software would be helpful to others in the field. Which portion of the software was used? The General Analysis or

© 2023. Published by The Company of Biologists under the terms of the Creative Commons Attribution License (https://creativecommons.org/licenses/by/4.0/).

AI? For segmentation several steps are involved that require user input but are not mentioned i.e. noise removal, intensity thresholding, object analysis and possibly other steps.
c. Calculation of mitochondrial metrics including the mitochondrial complexity index, which is defined in the results section but how this is calculated is not mentioned in methods.
d. How was TMRE quantified in Figure 4?

2. Membrane potential and cellular respiration
a. The Seahorse technology is very powerful but also can be misleading. The authors nicely complement their Seahorse data with O2K measurements to determine the impact of ETC components from Drp1 mutations. This is very well done. I did have a concern about the Seahorse data though. Figure 6E Seahorse control experiments gives maximal respiration rate that differs greatly from what is shown in 3A for WT and G32A. In 3A it appears that maximal respiration is not significantly different between any of the genotypes. Here in 6E they are quite different and appear to be statistically significant, which should be calculated and differences discussed.
b. Nice use of etomoxir, a CPT1 inhibitor, to probe the ability of patient cells to metabolize FA. The differences between R403C and G32A in the Seahorse experiments are surprising and I wonder to what extent they rely on the one extremely high data point that appears to be an outlier.
c. Analysis of TEM images show that patient cells have altered cristae morphology (Fig 5) but this does not appear to correlate with TMRE staining that indicates increased polarization across the inner membrane, which should be clarified.

Other Comments
3. Although most patients with Drp1 variants present with EMPF1, it is clear not all patients develop these symptoms, especially those with point substitutions in the GTPase domain. For example, Drp1 E2A and A192E (Gerber et al. 2017) presented solely with visual impairments. Similarly, in Whitley et al. (2018), G32A presented with optic atrophy and nystagmus in addition to the neurologic complications. Can the authors expand on their thoughts on the potential link to ophthalmic complications?

4. The authors should consider discussing what is known about G32A and R403C from prior studies. E.g. G32A is a conserved Gly in the GXXXXGK – Walker A/P loop. It is not invariant, though, as EF-G in E. coli has an G to A substitution. Interesting that this is not tolerated in Drp1. R403 is at the tip of the stalk and was known to mediate oligomerization in MyxA and dynamin leading to it being mutated in the Drp1 structure solved by Daumke and co-workers. These details are not necessary for the current study but some mention of these prior findings for context are welcome in context of their findings.
E.g. why does a mutation within the GTPase domain have such a large impact on downstream protein phosphorylation within the variable domain?

5. Many of the other pathological variants have altered assembly states (e.g. A395D). Perhaps a comment on this wrt to what is known about G32A and R403C?

6. Figure 3: including arrows indicating when each compound was added during the Seahorse assay may make this figure easier to understand for those who are less familiar with the technique.

7. How many total cells (and replicates) were used for the TEM imaging?

8. Do the pathological Drp1 variants have impaired peroxisomal localization? This could be very informative if certain mutations impact localization to only one organelle.

9. Were there any accompanying changes in fatty acid levels in the patients with G32A or R403C? If these studies were performed, they should be mentioned in relation to the gene expression findings.

10. Figure 7: “stalled fission” is not centered in the box in the model figure.

11. Supplemental figure 4: I would recommend the authors consider using a different order of their example scoring images (clockwise, left to right, etc).

© 2023. Published by The Company of Biologists under the terms of the Creative Commons Attribution License (https://creativecommons.org/licenses/by/4.0/).

12. The Authors are missing citations for a number of papers describing pathological Drp1 variants including: Díez et al. (2017), Batzir et al. (2019), Verrigni et al. (2019), Gerber et al. (2017), Yoon et al. (2014), Hogarth et al. (2018), Wei et al (2021), Nolden et al. (2022), Vanstone et al. (2018), and Zaha et al. (2016)

13. What isoform is the numbering in?

14. Lines 182-185 in the results section This is reported in text as looking at only p616Drp1 recruitment, but the figure is all Drp1? Clarify.

15. Cite the Frank et al 2001 Dev Cell paper when discussing Drp1 and delay of apoptosis. This was the seminal paper that recognized a connection between fission and apoptosis.

16. Lines 203-204 Etoposide and STS should be defined as well as their rational for use.

17. Line 273. “P” might need to be “Pal”.

18. As the authors point out, the hyperpolarization of the mitochondrial inner membrane by TMRE could arise from ATPSYnthase running in reverse, which is readily tested upon the addition of oligomycin which would have been a nice addition. Along these lines, the measurement of oxidative stress might be useful in future studies.

19. Figure 7 legend should indicate that this is live cell imaging and.

20. “peripheral fission” should be more clearly defined.

21. Is the 2nd row of Fig 6A and enlargement of 1st row? If so, indicate what regions were enlarged with a box in 1st row.

First revision

<u>Author response to reviewers' comments</u>

We thank the Reviewers of Journal of Cell Science for their time and feedback on our manuscript. As a result, the manuscript has been substantively recrafted with additional data and clarifications. Below you can find a detailed response to all reviewers’ comments.

Reviewer 1 Advance Summary and Potential Significance to Field:

In this manuscript by Gama and colleagues, the authors utilize patient-derived fibroblasts to functionally characterize the mitochondrial, peroxisomal, and metabolic defects associated with two disease-associated heterozygous mutations in the gene encoding for the mitochondrial fission protein Drp1. The authors confirmed previous analysis of these Drp1 mutations (G32A and R403C), which have been shown to compromise Drp1 function in mitochondrial fission and act in a dominant-negative manner. The authors extend the analysis of Drp1 mutation, for example, to demonstrate that they affect protein stability of mitofusins, mildly affect cristae morphology, lead to resistance against apoptosis induction, and cause altered peroxisomal morphology. The authors also perform metabolic analysis of these patient-derived lines and find reduced mRNA expression of two beta-oxidation pathway genes and potential differential beta-oxidation utilization.

Reviewer 1 Comments for the Author:
The work is generally well performed and the imaging is of high quality. However, most of the observed findings are consistent with previously published characterization of these specific patient mutations and/or are phenotypes already associated with loss of Drp1 function. For example, several studies have done seahorse assays on Drp1 KD/KO cells and fatty acid oxidation utilization has also been evaluated (for example, King et al., JBC, 2021). The observations here

© 2023. Published by The Company of Biologists under the terms of the Creative Commons Attribution License (https://creativecommons.org/licenses/by/4.0/).

(such as the reduction of mRNA levels of two beta-oxidation pathway genes), while somewhat interesting, are not followed up in depth. Thus, while there is no doubt that the findings of the manuscript are worthy of publication, the motivation for the study is not completely clear and there is not significant enough conceptual advance to recommend publication in JCS.

We thank Reviewer 1 for their perspective. We have included new data and rewritten several sections of the manuscript to clarify the motivation of the study. We want to convey the critical importance of these results for our understanding of the pathology of EMPF1, but also to provide additional insight into the consequences of abnormal mitochondrial and peroxisomal fission for cellular homeostasis. While there have been extensive publications on DRP1 KD/KO cells, very little is known about the molecular phenotypes caused by patient mutations in DRP1.

1. The examination of beta-oxidation capacity in cells defective of mitochondrial fission is potentially interesting, though underexplored. What does it mean that there is reduced mRNA expression (20-50% lower) of CPT1 or ACADM? Does this correspond to lower protein level? For example, in the authors' analysis, they mentioned that they found lower mRNA expression for MICOS subunits that was not observed to correspond to protein stability defects though this data was not shown. Is fatty acid uptake capability diminished or are beta-oxidation rates altered? The authors did a Seahorse assay on cells treated with etomoxir and found patient lines had different maximal ATP production, but the mechanistic basis for this is lacking. How do these defects relate to the patient disease phenotypes?

Reviewer 1 brings an excellent point. An extensive exploration of the mechanisms by which fission affects both mRNA and protein expression level of critical enzymes would be interesting, particularly if it can be coupled to functional insight. Regarding the Etomoxir studies (New Supplementary Figure 5 – Long Chain Fatty Acid Oxidation Stress Test), the cells are cultured in media containing 0.5 mM glucose, 1.0 mM glutamine, and 0.5 mM XF L-carnitine, and during the assay, the cells are switched to media containing 2 mM glucose, 0.5 mM L-Carnitine, and Palmitate. This media composition pushes the cells towards reliance on fatty acid oxidation. Under these conditions, it appears that both G32A and R403C patient cells have a lower maximal respiration when they are forced to rely on fatty acid oxidation. These data may be attributed to changes in CPT1 expression and mitochondrial/peroxisomal morphology. We have now clarified this aspect in the text and figure legend. We agree with Reviewer 1 about the importance of further exploring the mechanistic details of these observations. However, this would require extensive additional time beyond the allowed revision time because these mechanistic details should be examined in an isogenic background and in specific cell types relevant to phenotypes seen in patients—for example, neural progenitor cells. These experiments are currently ongoing in our laboratory.

2. Are the alterations in metabolic capacity of patient lines rescued by simultaneous depletion of fusion machinery? The authors note in the discussion that this could be used as a tool to uncouple the metabolic defects of peroxisomal shape alteration from those of mitochondrial hyperfusion. The manuscript would be strengthened by evaluating this idea.

We agree that this step is of critical importance. However, there are some technical limitations. While knocking down MFN2 decreases the hyperelongation of the mitochondria in mutant cells, the mitochondrial dynamics are still impaired. Another important variable is that MFN2 downregulation also has an effect in control cells, altering their mitochondrial dynamics. These results suggest that a fine-tuned balance needs to be achieved to differentiate the metabolic defects of peroxisomal shape alteration from those of mitochondrial hyperfusion. To investigate this in depth, we started a collaboration with Dr. Evripidis Gavathiotis who recently published the design of small molecule inhibitors/activators of MFN2 (PMID: 35798717). In our follow up studies, we aim to determine the potential of pharmacologically inhibiting mitochondrial fusion to restore mitochondrial dynamics in EMPF1 using induced pluripotent stem cell models systems, and to explore in detail the contribution of the peroxisomal alteration to the pathophysiology of EMPF1.

3. The section of the manuscript on mitochondrial respiratory activity was technically dense and made confusing by the use of non-standard acronyms (P + M, Pal, Rot). The manuscript would be strengthened by a clearer explanation of the rationale for such thorough analysis and how such

© 2023. Published by The Company of Biologists under the terms of the Creative Commons Attribution License (https://creativecommons.org/licenses/by/4.0/).

results informed follow up experiments. For example, what do the differences observed between R403C and the G32A mutation mean? How does this correspond to patient phenotypes?

We thank Reviewer 1 for bringing this issue to our attention. We agree that this section is technically dense. Considering this comment, we have made some changes, including reducing the number of abbreviations used within the results section of the manuscript and increasing the rationale of the experiments. The abbreviations we were using were defined on the BioBlast website by Dr. Gnaiger, who founded the Oroboros Instruments company, so we kept them only in the figure and provide explanation in figure legends.

Resource: https://wiki.oroboros.at/index.php/MitoPedia:_Substrates_and_metabolites.

The data from the Seahorse suggested that there may be a mild decrease in OxPhos that is compensated by an increase on glycolysis. The benefit of the O2K method is that it allows us to measure the in-cell activity of each ETC complex with defined substrates to better discern specific changes in the ETC that would explain the mild changes observed with the Seahorse. In the end, the changes were not dramatic for these cells, suggesting that there are cellular compensations that have happened to sustain OxPhos, which is consistent with the unchanged ATP levels. To start getting at the potential metabolic rewiring that cells with abnormal fission may be undergoing, we also performed a metabolomics analysis of control and mutant fibroblasts. These analyses revealed that patient cells have aberrant purine and pyrimidine biosynthesis, as well as changes in the methionine cycle.

With regards to the relationship to patient phenotypes, this is a tougher question to address. These cells are remarkably robust, and there are likely adaptive mechanisms that allow fibroblasts to be bioenergetically competent. Even with the relatively mild changes in ETC function, the cells grow a little slower, which may highlight the importance of these defects. Moreover, there are likely additional roles that mitochondria play in the cell that are impacted by the limitations in mitochondrial fission, (i.e., signaling, mitophagy, etc.) and experiments to measure changes with these systems would be fascinating to address but beyond the scope of this manuscript. When considering patient phenotypes, these mutations lead to severe debilitating neurodevelopmental issues, which may be more tissue specific and hard to replicate with isolated fibroblasts. Our team is currently working on modeling these mutations in iPSC-derived systems to address the physiological effects of these mutations in neural cells.

4. The title of the paper is misleading as cristae morphology, while slightly altered, is not grossly affected in the Drp1 patient lines. Cristae are certainly affected by loss of Drp1, but whether the process of fission itself is required for their maintenance is not directly evaluated. Additionally, effects of Drp1 KD on cristae morphology have been previously reported (Ban-Ishihara et al., PNAS, 2013).

We completely agree with Reviewer 1 about the title of the paper. We have now modified it to more directly summarize the main message of our study. We have also cited the Ban-Ishihara et al study.

5. The authors make the point that mitochondrial morphology is corrected by MFN2 depletion while peroxisomal morphology is not, though images are not shown of the peroxisomes.

We have now clarified that the images of peroxisomes, marked by PMP70, are included in Figure 7C, together with the quantification (Supplementary Figure 6B).

6. Hyperpolarization of mitochondria as assessed by TMRE staining should be normalized to a non-membrane potential dependent mitochondrial label such as mitotracker green.

Based on this comment, which was also suggested by Reviewer 2, we repeated these experiments and imaged TMRE with MitoTracker green. The new data are included in new Figure 4. Furthermore, quantification of the TMRE signal relative to MitoTracker is included in Figure 4 as well. To note, we noticed that over time the mutant cells may be more sensitive to the time lapse acquisition.

© 2023. Published by The Company of Biologists under the terms of the Creative Commons Attribution License (https://creativecommons.org/licenses/by/4.0/).

7. Line 380 – the authors refer to siRNA of MFN2 as deletion. Also, no Western blots are shown of MFN2 (or MFN1) in this knockdown.

We have now corrected “deletion” and replaced it with “downregulation.” The Western blots for MFN2 are now included in Supplementary Figure 6A.

Reviewer 2 Advance Summary and Potential Significance to Field:

In their manuscript Robertson et al. perform detailed and high-quality characterization of the mitochondrial network and metabolism in fibroblasts derived from patients harboring de novo missense mutations in the DNM1L gene. DNM1L encodes DRP1, a protein that is essential for executing both mitochondrial and peroxisomal fission by mediating outer membrane constriction. Both mutations studied (G32A and R403C) are heterozygous in patients and have been reported to exert a dominant-negative effect on mitochondrial fission when overexpressed on the background of wild-type DRP1. In keeping with previous studies, the authors observe a hyperfused mitochondrial network in both G32A and R403C patient cell lines by SIM, block-facing scanning EM and transmission EM. TEM image analysis reveals that the hyperfused mitochondria have fewer and more irregular cristae. Additionally, SIM imaging shows a hyperfused peroxisomal network. The authors find a number of functional consequences of the mitochondrial hyperfusion phenotype: a delayed apoptotic response to stress, a decrease in the ETC coupling efficiency, an increase in glycolysis rate and interestingly hyperpolarization of the inner mitochondrial membrane. Both mitochondrial and peroxisomal hyperfusion phenotypes may contribute to the decreased dependance of patient fibroblasts on fatty acid oxidation. Although mitochondrial fusion machinery proteins Mitofusin 1 and Mitofusin 2 are already downregulated in patient fibroblasts, further depletion of Mitofusin 2 by siRNA rescued the mitochondrial fusion phenotype.
Compared to previous publications characterizing dominant-negative DRP1 mutations, this study provides an in-depth characterization of consequences of mitochondrial hyperfusion on cellular metabolism. Although patient-derived iPSC neurons would be more relevant to study EMPF1 pathology, the careful dissection provided here will provide a solid basis for future studies. Some revisions are necessary to ensure that the data reported justifies the conclusions drawn.

We thank Reviewer 2 for the positive comments about our study. We hope that the new data will strengthen our conclusions, and most importantly, as mentioned by Reviewer 2, that it can provide the basis for additional studies. We have now completed additional experiments, which have opened interesting avenues of investigation.

Reviewer 2 Comments for the Author:
Major points:

1) The authors provide a model (Figure 7), in which dominant-negative DRP1 mutants do not prevent recruitment of DRP1 to the mitochondrial surface, but instead result in stalled fission intermediates where DRP1 is present, yet unable to facilitate complete membrane fission. In Figures 2C and 2D the authors use immunofluorescence to show no difference in the number and volume of pS616-DRP1 mitochondrial puncta between control and patient-derived fibroblasts. (I assume there is an error in the figure legend here which should be addressed.) In the main text description of this figure (line 187-188) the authors state that ‘in the patient fibroblasts this interaction appeared less transient’. It is not clear to me how this conclusion can be made from the fixed-cell immunofluorescence data provided. In order to support their claim, the authors should consider time-resolved imaging of co-localizing puncta in cells overexpressing DRP1 variants as in Figures 1F and 1G. Whitley et al., 2018 report that while overexpression of GFP- fusion constructs of both the G32A and R403C variants result in a mitochondrial hyperfused phenotype, overexpressed GFP-DRP1-G32A remained entirely cytosolic and was not recruited to mitochondrial puncta. These findings should be discussed in light of the proposed model.

We thank Reviewer 2 for this insightful comment. We have now corrected the figure 2C-D legend to state that we measured pS616-DRP1. We agree that our conclusion of stalled fission was premature and lacked supporting data. We address this possibility in two different ways: 1) We performed live-cell imaging using MitoTracker green and mCherry-DRP1 constructs as suggested by Reviewer 2. While all forms of DRP1 were recruited to the mitochondria, the live cell imaging experiments revealed an overall decrease in the time that the mutant forms of DRP1 spend at the

© 2023. Published by The Company of Biologists under the terms of the Creative Commons Attribution License (https://creativecommons.org/licenses/by/4.0/).

mitochondrial membrane (New Supplementary Fig. 2). Since this defect of the mutant proteins was only detected in the live cell imaging experiments, the possibility that this phenotype may only become evident in conditions of stress (phototoxicity during live cell imaging in this case), can't be excluded. It would be informative to determine in the future if the mutant dimers are recruited to the mitochondria and sequester the WT forms of the protein, explaining the dominant negative effects. Establishing the kinetics of association and dissociation of the mutant and WT forms of DRP1 in both homeostatic and stressed conditions could provide additional insight into the mechanism behind these phenotypes. 2) In experiments not included in the manuscript, we also performed proximity ligation assays to determine whether there are any alterations in the association of DRP1 with the effector proteins. We found that there indeed seems to be increased association between DRP1 and FIS1, but no other adaptors, in the mutant cells (Figure 1 for reviewers only). These results are intriguing, but their interpretation is not straightforward since we did not assess for binding to adaptors at the peroxisomes, and in both PLA and pS616-DRP1 recruitment, we are unable to distinguish the signal from WT DRP1 or mutant DRP1. The data in Figure 1F-G and new Figure 3 at time 0 in the live-cell imaging experiments suggest that both WT and mutant DRP1 have similar capacity to be recruited to the mitochondria, but whether the stalled fission phenomenon is due to different kinetics of association or dissociation is less clear. We decided to delete the "less transient" statement from the manuscript and introduced the results explained above.

2) The authors argue that the reduction of Mitofusin 1 and 2 levels are the result of protein destabilization associated with the lack of mitochondrial fission without checking transcript levels. To support their conclusion, it is necessary to check Mitofusin transcript levels by qPCR, as has been done for other mitochondrial proteins assessed in this study.

We have now included qRT-PCR results of MFN1 and MFN2 (Supplementary Figure 2E) and found that the overall transcript levels of both *MFN1* and *MFN2* are reduced, but this decrease only reaches significance for *MFN2* in the R403C. Based on these results, we edited the manuscript to reflect these new data.

Minor points:

1) It is not clear whether control fibroblasts match characteristics of the patients from whom the G32A and R403C lines were derived. The authors should discuss the source for all three described cell lines in the Materials and Methods section.

We thank the reviewer for pointing out this oversight. This information is now included in the Materials and Methods section under Cell Culture to clarify that the patients are age-matched, and the cells are passage-matched.

2) To aid interpretation of Figure 3 as well as the main text description of O2K respiration measurements, it would be helpful for the authors to include representative traces for both protocols in a supplementary figure, indicating at what points values are taken for comparison in the main figure. Could the authors clarify why there are different trends in the Intact Cellular Respiration rate at the beginning of O2K Protocol 1 vs Protocol 2? The discussed elevated ICR rate observed for the R403C line in Protocol 2 before and after Malate addition does not seem to be reproduced in Protocol 1.

We have now included representative traces for both protocols (Supplemental Figure 3). It is true that there is variability in the ICR rates between both protocols which could explain why we don't see a significant difference with Protocol 1. Experimentally, we don't have a reasonable explanation for the difference we see with Protocol 2, other than technical variability between both experiments either due to user or state of the cells.

We also see variability depending on the availability of fatty acids. It appears that both G32A and R403C cells have a lower maximal respiration when they are forced to rely on fatty acid oxidation. However, this does not explain the difference in ICR between both protocols.

© 2023. Published by The Company of Biologists under the terms of the Creative Commons Attribution License (https://creativecommons.org/licenses/by/4.0/).

3) The mitochondrial hyperpolarization in both patient cell lines is interesting, however quantification of this phenomenon is necessary. Would it be possible to show quantification across all cells in the reported independent experiments?

We agree completely with this point. In order to quantify the degree of mitochondrial membrane polarization, we repeated the experiments to determine fluorescence intensity of TMRE relative to MitoTracker green. These data are now included in New Figure 4.

4) Figures 7C and 7D are not referred to in the main text – I believe they should be referred to in place of Figure 7B on line 381.

We thank Reviewer 2 for finding this error. This was fixed in the revised version of the manuscript.

Reviewer 3 Advance Summary and Potential Significance to Field:

In Robertson et al., the authors aimed to determine the cellular consequences of two prior reported single nucleotide Drp1 variants that cause encephalopathy due to defective mitochondrial and peroxisomal fission (EMPF1) in patients, one in the GTPase domain (G32A) and one in the stalk domain (R403C). To accomplish this, the authors used a combination of live cell fluorescence and super-resolution microscopy on both patient-derived fibroblasts and control fibroblasts undergoing transient transfection, transmission electron microscopy to evaluate mitochondrial cristae structure, cellular metabolism measurements, and both gene and protein expression quantification. In doing so, the authors perform a thorough characterization of the two EMPF1-associated variants and perform one of the most thorough metabolic analyses to date for pathological Drp1 variants. In fact the manuscripts presents many firsts in the field with respect to Drp1 pathological variants (first detailed report of how pathological variants of Drp1 impact cellular respiration, first use of super-resolution, first investigation of Drp1 Ser616 phosphorylation in pathological variants, first counting of fission and fusion events, and others. Further, the authors expand on the prior studies in which these variants were initially identified, concluding that not only is mitochondrial fission disrupted, but so is the frequency of fission events, mitochondrial membrane potential, and cell response to apoptotic stimuli. Further, peroxisomal fission, and levels of peroxisomal proteins involved in fatty acid metabolism, are dysregulated.

Overall, the experiments in this study are extremely thorough and of high quality. In addition, the authors are cognizant of the limitations of the study. The manuscript would be improved by more of a discussion on how their findings fit in the context of the growing number of pathological Drp1 variants that have been identified and characterized. Further, it is interesting that mutations in two different domains can lead to similar functional impairments, albeit with different clinical presentations and apparent mechanistic causes. Other mutations within the GTPase and stalk domain have also been characterized, typically with vastly different constellations of patient symptoms and disease severity. Given this, it would have been interesting to have more insight into why the authors thought these differential effects might exist.

We appreciate Reviewer 3's comments and the positive feedback on our study. We agree that additional discussion and clarifications would enhance the support for the conclusions drawn by our study. We have now performed additional experiments, expanded the discussion section, and considered the points brought by Reviewer 3.

Reviewer 3 Comments for the Author:

Main comments

1. Insufficient details in many places

We apologize for this oversight. In response to this comment by Reviewer 3, we have now prepared an extended version of the methods and included it as supplemental information.

a. Throughout methods section, details on data collection are scant (e.g. z-slices for Fig 7).

© 2023. Published by The Company of Biologists under the terms of the Creative Commons Attribution License (https://creativecommons.org/licenses/by/4.0/).

The size and number of Z-slices for all microscopy are now included in the methods section under image acquisition. All figure legends are updated to indicate that the representative image is a max-intensity projection.

b. Details on microscopy data analysis throughout are lacking in places. E.g. how mitochondrial volume, surface area, and major axis length are determined with Nikon Elements software would be helpful to others in the field. Which portion of the software was used? The General Analysis or AI? For segmentation, several steps are involved that require user input but are not mentioned i.e. noise removal, intensity thresholding, object analysis and possibly other steps.

The Nikon elements GA3 workflow is now included in the methods. [Editorial note: All details are not included in the Methods section. The Nikon elements GA3 workflow will be available upon request.]

c. Calculation of mitochondrial metrics including the mitochondrial complexity index, which is defined in the results section but how this is calculated is not mentioned in methods.

This measurement is now explained in the methods section of the paper.

d. How was TMRE quantified in Figure 4?

In order to quantify the degree of mitochondrial membrane polarization, we repeated the experiments to determine fluorescence intensity of TMRE relative to MitoTracker green (New Figure 4). Cells were labeled with 100nM MitoTracker Green for 30 minutes followed by 500nM TMRE for 10 minutes. After one wash with media, cells were imaged every 10 seconds for 3 minutes.

2. Membrane potential and cellular respiration. The Seahorse technology is very powerful but also can be misleading. The authors nicely complement their Seahorse data with O2K measurements to determine the impact of ETC components from Drp1 mutations. This is very well done. I did have a concern about the Seahorse data though. Figure 6E Seahorse control experiments gives maximal respiration rate that differs greatly from what is shown in 3A for WT and G32A. In 3A it appears that maximal respiration is not significantly different between any of the genotypes. Here in 6E they are quite different and appear to be statistically significant, which should be calculated and differences discussed.

We completely agree with this point brought by Reviewer 3. The difference in basal and maximal respiration for control (WT) between Figures 3 and 6 is due to substrate limited media used in Figure 6. For Figure 3 (Mito Stress Test), the cells are cultured in their usual media containing 2 mM glutamine and 5.55 mM glucose, and during the assay, the cells are in media containing 1 mM pyruvate, 2 mM glutamine, and 10 mM glucose. For Figure 6 (Long Chain Fatty Acid Oxidation Stress Test), the cells are cultured in media containing 0.5 mM glucose, 1.0 mM glutamine, and 0.5 mM XF L-carnitine, and during the assay, the cells are in media containing 2 mM glucose, 0.5 mM L-Carnitine, and Palmitate.

The second point about differences in maximal respiration between the genotypes in Figure 6 is correct. We have now added these results to the figure and results. Given the differences in substrate composition, it appears that both G32A and R403C patient cells have a lower maximal respiration when they are forced to rely on fatty acid oxidation. This is not totally unexpected given the reduction in CPT1 expression and changes in peroxisomal morphology.

b. Nice use of etomoxir, a CPT1 inhibitor, to probe the ability of patient cells to metabolize FA. The differences between R403C and G32A in the Seahorse experiments are surprising and I wonder to what extent they rely on the one extremely high data point that appears to be an outlier.

We performed ROUT outlier testing, which uses both robust regression and outlier removal to determine whether there are statistical outliers in the data. Even with very strict parameters, the data don’t appear to have any statistical outliers.

© 2023. Published by The Company of Biologists under the terms of the Creative Commons Attribution License (https://creativecommons.org/licenses/by/4.0/).

The Seahorse traces with etomoxir for each individual n are now included in Fig. 2 for Reviewer's only. The data show that etomoxir increases maximal respiration for the R403C cells in one replicate only. In the other replicates, etomoxir simply doesn't decrease maximal respiration. Since this dramatic increase is only seen in one replicate, we can only conclude that etomoxir doesn't decrease maximal respiration, which we believe is a fair conclusion from the data.

c. Analysis of TEM images show that patient cells have altered cristae morphology (Fig 5) but this does not appear to correlate with TMRE staining that indicates increased polarization across the inner membrane, which should be clarified.

We thank the reviewer for pointing out this point of confusion. Although cristae morphology is significantly altered, Seahorse and O2K analysis both indicate that patient cells are capable of basal respiration similar to control. However, patient cells have an increased proton leak which we could speculate may increase the mitochondrial membrane potential. Additional studies are needed to confirm this claim. We will clarify this point in the results and discussion.

Other Comments

3. Although most patients with Drp1 variants present with EMPF1, it is clear not all patients develop these symptoms, especially those with point substitutions in the GTPase domain. For example, Drp1 E2A and A192E (Gerber et al. 2017) presented solely with visual impairments. Similarly, in Whitley et al. (2018), G32A presented with optic atrophy and nystagmus in addition to the neurologic complications. Can the authors expand on their thoughts on the potential link to ophthalmic complications?

OPA1, a protein involved in mitochondrial fusion, has been associated with ophthalmic complications as well. The relationship between mutations in DRP1 and OPA1 is very intriguing and argues for some shared phenotype caused by mitochondrial dysregulation. Although DRP1 and OPA1 preform opposite roles in regard to fission and fusion, they may both impact cristae morphology and therefore have a similar impact in patient outcome. Interestingly, many patients with DRP1 mutations also present with spasticity and muscle tone abnormalities. Both motor neurons and the neurons of the optic nerve have extremely long axons. Therefore, these cells may be especially sensitive to defects in mitochondrial morphology.

4. The authors should consider discussing what is known about G32A and R403C from prior studies. E.g. G32A is a conserved Gly in the GXXXXGK – Walker A/P loop. It is not invariant, though, as EF-G in E. coli has an G to A substitution. Interesting that this is not tolerated in Drp1. R403 is at the tip of the stalk and was known to mediate oligomerization in MyxA and dynamin leading to it being mutated in the Drp1 structure solved by Daumke and co-workers. These details are not necessary for the current study but some mention of these prior findings for context are welcome in context of their findings. E.g. why does a mutation within the GTPase domain have such a large impact on downstream protein phosphorylation within the variable domain?

We appreciate the insight into the structural properties of DRP1. Our collaborator, Jason Mears, has studied these mutations in his laboratory using recombinant proteins. As mentioned by Reviewer 3, the G32 position in DRP1 is a highly conserved site, but the high degree of conservation has not been fully explained in the literature. It is the first residue in the P loop, and the glycine is important because it breaks the secondary structure to start the loop. In fact, the sequence in the loop is not highly conserved (GxxxxGK) because different families of NTPases bind and hydrolyze the nucleotide phosphates differently.

Mutations in distinct domains would be predicted to have different effects on Drp1 function. Specifically, the G32A mutation is in the highly conserved P loop (Saraste et al, TiBS, 1990 – v. 15, Issue 11, pp. 430-434), which would be predicted to affect the catalytic activity of the protein. The R403C mutation is in a loop that is important for protein self-assembly (Frolich et al paper in EMBO, 2013 and Gao et al, Immunity, 2011) since this region was mutated (poly-alanine mutants) in both Drp1 and MyxA to limit self-assembly and allow for crystallization.

© 2023. Published by The Company of Biologists under the terms of the Creative Commons Attribution License (https://creativecommons.org/licenses/by/4.0/).

With regard to the phospho 616 residue, it is hard to speculate in the discussion because we cannot distinguish between WT and mutant DRP1—a limitation of our experiments. The G32A mutation is still going to be assembly competent, and the residence time and localization with mitochondria would be increased because hydrolysis is likely impaired. A way to then temper the increase in co-localization would be to suppress the activating S616 phosphorylation. The R403C mutation is likely defective in assembly, so DRP1 recruitment to mitochondria would be impaired for this mutant, so S616 phosphorylation is still needed to promote recruitment. We are hesitant to imply this model without some way to distinguish between the WT vs mutated protein recruitment to the OMM.

Based on our collaborator's unpublished data using recombinant proteins, the G32A mutant DRP1 is defective in GTPase activity, and it has no issue binding and tubulating lipid templates (i.e. it can assembly the same as WT). Conversely, the R403C mutant protein is assembly defective. It is stuck as a dimer and cannot build helical polymers because of the mutation in that conserved loop at the base of the stalk. The live cell imaging suggests that a proportion of R403C is recruited to mitochondria, but it appears to rapidly dissociate (ring-like structures can be seen in the cytosolic pool). Thus, these two mutations exhibit the expected defects based on their positions in conserved catalytic and assembly loops, respectively.

5. Many of the other pathological variants have altered assembly states (e.g. A395D). Perhaps a comment on this wrt to what is known about G32A and R403C?

The A395D mutant was defective in self-assembly (Chang et al, JBC, 2010). The caveat with the A395D report is that this protein had a tag on. A more recent report described mutations at G363D and G401S, and both of these mutations were dimer-limited (i.e. assembly defective). The G401S is in the same loop as R403C, which was mutated in the crystal structure studies of DRP1 (residues 401-404, GPRP to AAAA) (Nolden et al, 2022, Life Sci Alliance).

6. Figure 3: including arrows indicating when each compound was added during the Seahorse assay may make this figure easier to understand for those who are less familiar with the technique.

Figure 3A has been modified for clarity.

7. How many total cells (and replicates) were used for the TEM imaging?

For TEM imaging, 7 cells were imaged per biological replicate. 3 independent experiments were completed for a total of 21 cells.

8. Do the pathological Drp1 variants have impaired peroxisomal localization? This could be very informative if certain mutations impact localization to only one organelle.

We agreed with Reviewer 3 that this was of great interest. Thus, during the review process, we performed this experiment and found interesting differences between the two mutant cells. When examining the recruitment of total DRP1 to the peroxisomes, R403C cells appear to have a more dramatic impairment in DRP1 recruitment to these organelles, confirming the defect in assembly. These data are now included in Supplementary Figure 5A-B.

9. Were there any accompanying changes in fatty acid levels in the patients with G32A or R403C? If these studies were performed, they should be mentioned in relation to the gene expression findings.

Unfortunately, of the 39 EMPF1 cases reported to date, only a few of them report metabolic analysis related to very long-chain fatty acids (VLCFAs), thus no conclusions can be drawn with such low sample number. Whitley et al. 2018 reported that the patient harboring the G32A mutation has elevated very VLCFAs. Ryan et al., 2018 revealed normal VLCFAs levels in the R403C mutant. A report by Lhuissier et al., 2022 showed normal levels of VLFAs in a patient with a mutation in the GTPase domain.

© 2023. Published by The Company of Biologists under the terms of the Creative Commons Attribution License (https://creativecommons.org/licenses/by/4.0/).

Hyperlactacidemia, on the other hand, has been reported in 4 cases of 12 patients with GTPase domain mutations and 13 cases of 20 patients with middle domain mutations.

10. Figure 7: “stalled fission” is not centered in the box in the model figure.

We apologize for the oversight in this figure. We have now modified the figure accordingly.

11. Supplemental figure 4: I would recommend the authors consider using a different order of their example scoring images (clockwise, left to right, etc).

We thank the reviewer for helping to clarify this figure. The figure was modified to orient the scoring in a clockwise manner.

12. The Authors are missing citations for a number of papers describing pathological Drp1 variants including: Díez et al. (2017), Batzir et al. (2019), Verrigni et al. (2019), Gerber et al. (2017), Yoon et al. (2014), Hogarth et al. (2018), Wei et al (2021), Nolden et al. (2022), Vanstone et al. (2018), and Zaha et al. (2016)

We thank reviewer for these suggestions. We have now included all the citations reporting patient variants.

13. What isoform is the numbering in?

The numbering correspond to variant 4.

14. Lines 182-185 in the results section This is reported in text as looking at only p616Drp1 recruitment, but the figure is all Drp1? Clarify.

We apologize for the confusion and mistake in labeling. The immunofluorescence images are indeed of pS616-DRP1. We have clarified the figure to match the text.

15. Cite the Frank et al 2001 Dev Cell paper when discussing Drp1 and delay of apoptosis. This was the seminal paper that recognized a connection between fission and apoptosis.

We have rectified this oversight and now include this reference in the revised version of the manuscript.

16. Lines 203-204 Etoposide and STS should be defined as well as their rational for use.

We have now included the definition and rationale for use of these compounds in this experiment.

17. Line 273. “P” might need to be “Pal”.

Based on reviewer comments, we have modified this section to include fewer acronyms and have replaced “P” with palmitoylcarnitine.

18. As the authors point out, the hyperpolarization of the mitochondrial inner membrane by TMRE could arise from ATP Synthase running in reverse, which is readily tested upon the addition of oligomycin which would have been a nice addition. Along these lines, the measurement of oxidative stress might be useful in future studies.

We thank the reviewer for these suggestions for future studies. We have now added a more thorough quantification of TMRE relative to MitoTracker. In future studies, we plan to examine the mechanism by which aberrant mitochondrial fission causes hyperpolarized membrane potential and if any treatments/interventions can reverse this phenotype. We performed a preliminary study to check whether oligomycin at the same concentration used in the Seahorse assay would affect mitochondrial membrane polarization, and we did not see an effect. A careful analysis in a dose and time-dependent manner may be needed coupling both readouts: ATP production and TMRE staining.

© 2023. Published by The Company of Biologists under the terms of the Creative Commons Attribution License (https://creativecommons.org/licenses/by/4.0/).

19. Figure 7 legend should indicate that this is live cell imaging

We apologize for this lack of clarity. This has been indicated in the figure legend and results section.

20. “peripheral fission” should be more clearly defined.

We thank the reviewer for the suggestion and have defined this term in the manuscript.

21. Is the 2nd row of Fig 6A and enlargement of 1st row? If so, indicate what regions were enlarged with a box in 1st row.

We have now indicated the area of enlargement with a box in the new version of Fig. 6, as well as added the information in the figure legend.

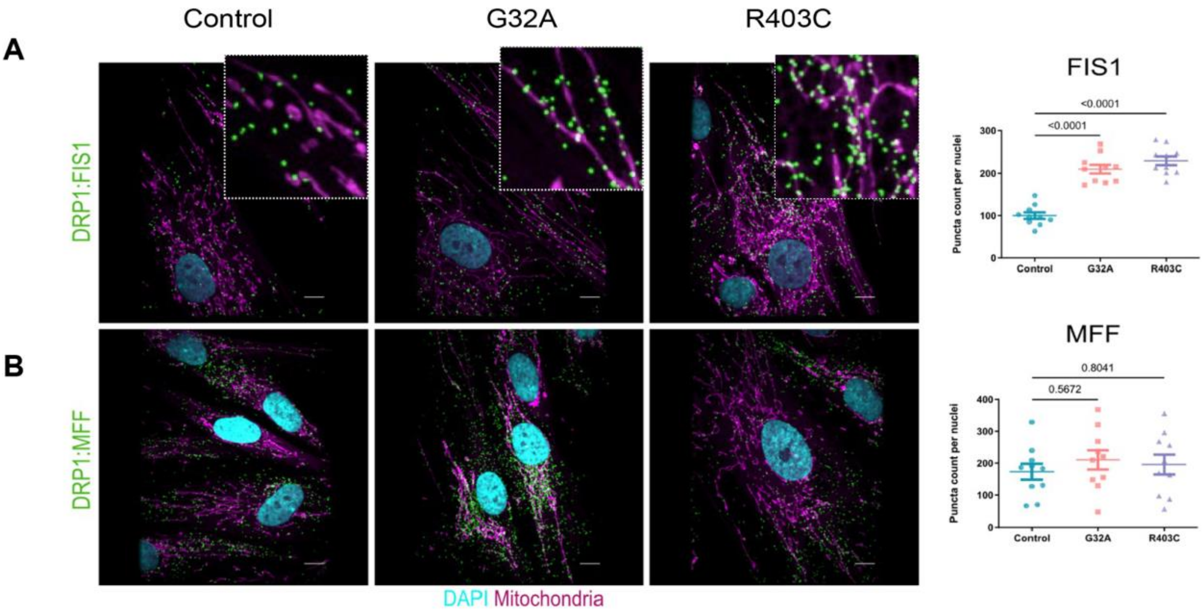

© 2023. Published by The Company of Biologists under the terms of the Creative Commons Attribution License (https://creativecommons.org/licenses/by/4.0/).

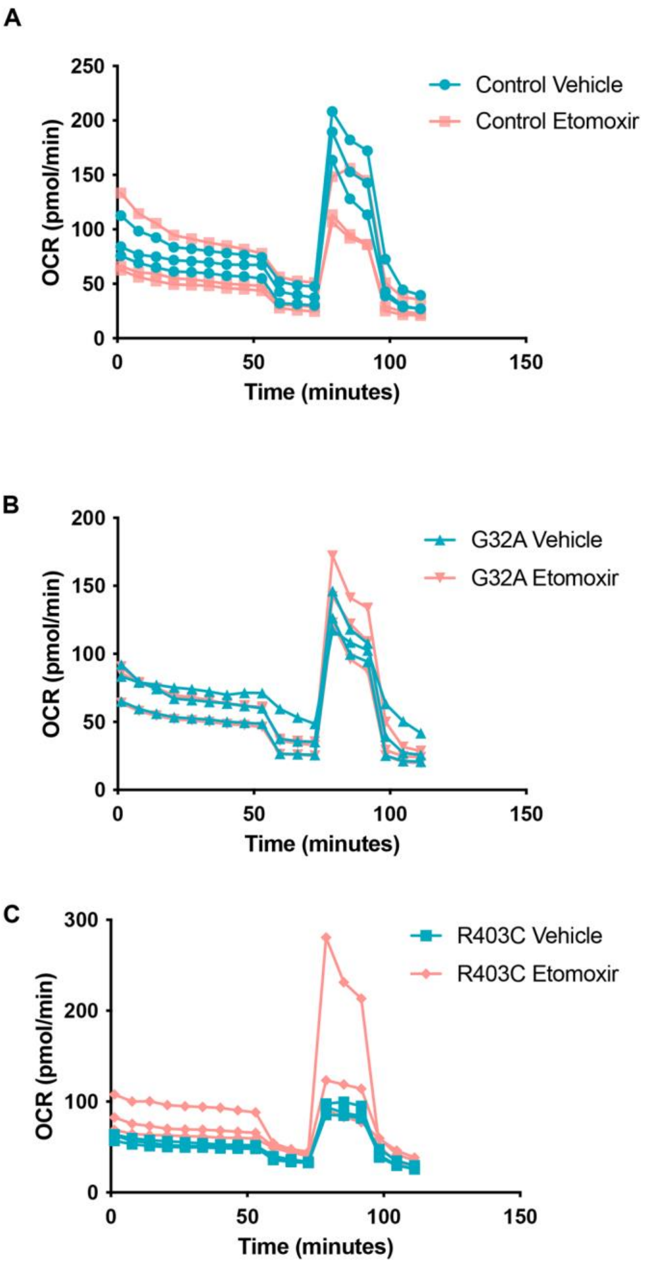

<u>Second decision letter</u>

MS ID#: JOCES/2022/260370

MS TITLE: DRP1 mutations associated with EMPF1 encephalopathy alter mitochondrial membrane potential and metabolic programs

AUTHORS: Gabriella L. Robertson, Stellan Riffle, Mira Patel, Caroline Bodnya, Andrea Marshall, Heather Beasley, Edgar Garza Lopez, Jianqiang Shao, Zer Vue, Antentor Hinton Jr., Maria S. Stoll, Sholto de Wet, Ram Prosad Chakrabart, Rensu P. Theart, Ben Loos, Navdeep Chandel, Jason A. Mears, and Vivian Gama
ARTICLE TYPE: Research Article

I am happy to tell you that your manuscript has been accepted for publication in Journal of Cell Science, pending standard ethics checks.

© 2023. Published by The Company of Biologists under the terms of the Creative Commons Attribution License (https://creativecommons.org/licenses/by/4.0/).
